# Vitamin D Supplementation and Allergic Diseases during Childhood: A Systematic Review and Meta-Analysis

**DOI:** 10.3390/nu14193947

**Published:** 2022-09-23

**Authors:** Qinyuan Li, Qi Zhou, Guangli Zhang, Xiaoyin Tian, Yuanyuan Li, Zhili Wang, Yan Zhao, Yaolong Chen, Zhengxiu Luo

**Affiliations:** 1 Department of Respiratory Medicine, Children’s Hospital of Chongqing Medical University, National Clinical Research Center for Child Health and Disorders, Ministry of Education Key Laboratory of Child Development and Disorders, Chongqing Key Laboratory of Pediatrics, Chongqing 400010, China; 2 Evidence-Based Medicine Center, School of Basic Medical Sciences, Lanzhou University, Lanzhou 730000, China; 3 Research Unit of Evidence-Based Evaluation and Guidelines, Chinese Academy of Medical Sciences (2021RU017), School of Basic Medical Sciences, Lanzhou University, Lanzhou 730000, China; 4 Chinese GRADE Centre, Lanzhou 730000, China; 5 Lanzhou University, An Affiliate of the Cochrane China Network, Lanzhou 730000, China; 6 Chevidence Lab of Child and Adolescent Health, Children’s Hospital of Chongqing Medical University, National Clinical Research Center for Child Health and Disorders, Ministry of Education Key Laboratory of Child Development and Disorders, Chongqing Key Laboratory of Pediatrics, Chongqing 400010, China

**Keywords:** vitamin D, asthma, allergic rhinitis, atopic dermatitis, children

## Abstract

We performed a systematic review and meta-analysis to investigate the effects of vitamin D (VitD) supplementation on children with allergic diseases. MEDLINE, Embase, Web of Science, the Cochrane library, and three Chinese databases were searched up to 15 August 2022. Randomized controlled trials (RCTs) comparing a VitD supplementation versus a placebo for children with allergic diseases were included. Thirty-two RCTs with 2347 participants were included. VitD supplementation did not reduce the risk of asthma exacerbations in children compared with placebo overall (risk ratio (RR) = 0.84, 95% confidence interval (CI): 0.65 to 1.08, *p* = 0.18), but reduced the risk of asthma exacerbation in children with baseline serum 25(OH)D of <10 ng/mL compared with placebo (RR = 0.48, 95% CI: 0.28 to 0.83, *p* = 0.009). VitD supplementation significantly reduced Scoring Atopic Dermatitis or the Eczema Area and Severity Index scores in children with atopic dermatitis compared with placebo (standard mean difference = −0.5, 95% CI: −0.87 to −0.12, *p* = 0.009). VitD supplementation also reduced the symptom-medication score in children with allergic rhinitis compared with placebo (mean (standard deviation): 43.7 (3.3) vs. 57.8 (4.4), *p* = 0.001). In conclusion, VitD supplementation did not reduce asthma exacerbation risk in children overall but may reduce asthma exacerbation risk in children with serum 25(OH)D concentration < 10 ng/mL. VitD supplementation reduces the severity of atopic dermatitis and symptoms of allergic rhinitis in children.

## 1. Introduction

Allergic diseases, mainly involving asthma, allergic rhinitis (AR), atopic dermatitis (AD), and food allergy, now affect approximately 20% of the global population [1]. These diseases impose a tremendous burden on individuals, their families, and societies by reducing quality of life and productivity at work or school and causing considerable economic costs [1]. The prevalence of allergic diseases has continued to increase over the past few decades, especially among children [2]. This could be partially explained by genetic susceptibility, but this is also related to environmental factors, such as vitamin D deficiency [3].

Vitamin D is a pleiotropic hormone [4]. In addition to regulating calcium and phosphorus metabolism, vitamin D has strong immunomodulatory effects [4]. Vitamin D can inhibit type 2 helper T cell (Th2 cell) function [5]. It also inhibits B cell proliferation and differentiation to plasma cells, resulting in the diminished secretion of immunoglobulin (Ig) E [6]. It has been well acknowledged that Th2 response plays a critical role in all allergic diseases [7]. Hence, the growing interest in the effect of vitamin D on allergic diseases has been generated.

Many observational studies have found that vitamin D deficiency was highly prevalent in children with allergic diseases [8,9,10]. However, it is unclear whether vitamin D supplementation can improve the outcomes of children with allergic diseases. The effects of vitamin D supplementation on certain allergic diseases, such as asthma and atopic dermatitis, have been discussed in several systematic reviews (SRs) [11,12,13,14]. However, the effects of vitamin D supplementation on all allergic diseases in children have not been examined. The common comorbidities and shared pathogenesis of allergic diseases have led to the need for a common therapeutic approach [15,16,17]. Therefore, a holistic overview of the treatment effect of vitamin D on childhood allergic diseases is necessary. Some of the previous SRs did not identify the individuals who might derive the greatest benefit from vitamin D supplementation and did not examine the optimal regimen of vitamin D [12,13,14]. To resolve the above issues, we performed an SR and meta-analysis to investigate the effects of vitamin D supplementation on childhood allergic diseases.

## 2. Materials and Methods

This SR was performed in accordance with the Cochrane Handbook [18]. We reported the results in accordance with the Preferred Reporting Items for Systematic Reviews and Meta-Analysis (PRISMA) statement [19]. This review has been registered on the PROSPERO (CRD42022299761).

### 2.1. Search Strategy

We searched MEDLINE, Embase, Web of Science, the Cochrane Library, China Biology Medicine (CBM), China National Knowledge Infrastructure (CNKI), and Wanfang Data databases for eligible studies from inception to 15 August 2022, without language restriction. Further details of the search strategy can be found in Table S1 in the Supplementary Materials. We also searched ClinicalTrials.gov, WHO International Clinical Trial Registry Platform, and Google.com for any relevant randomized controlled trials (RCTs). Searches were supplemented by handsearching through the reference lists of the included publications and previous SRs.

### 2.2. Eligibility Criteria and Study Selection

RCTs were assessed for eligibility criteria by two groups of experienced investigators (group 1 was QL and QZ; group 2 was ZW and YZ) who studied and evaluated these RCTs after careful consideration. Any discrepancy was resolved through consensus discussion or consultation with a third experienced investigator (ZL). The inclusion criteria were: (1) population: children aged 1 year to 18 years with allergic diseases such as asthma, allergic rhinitis (AR), atopic dermatitis (AD), food allergies, and anaphylaxis; (2) intervention: vitamin D supplementation; (3) control: placebo or no supplementation. We also allowed a maintenance dose of vitamin D (400 IU daily) as a control (compared with a high dose of vitamin D as an intervention) because some authors believe it was unethical to not provide vitamin D to the control group, who were suspected to have low serum 25(OH)D levels at enrollment [20]. We excluded publications that did not present research findings (e.g., narrative reviews, editorials, comments) or did not provide separate data for children.

### 2.3. Data Extraction

Two groups of investigators (group 1 was QL and QZ; group 2 was GZ, XT, and YL) extracted data in a blinded way. Data from each study were tabulated and checked by a third investigator (ZL) before inclusion in the analysis. The details of the data extraction are shown in Appendix S1 in the Supplementary Materials. The primary outcome for asthma was the number of children with one or more asthma exacerbations. Asthma exacerbation was defined as having at least one of the following: taking systemic corticosteroids for asthma exacerbation; an asthma-related emergency department (ED) visit or hospitalization, or both; taking short-acting β-agonists as quick-relief medication; or a physician-diagnosed exacerbation. The secondary outcomes for asthma include the number of children requiring systemic corticosteroids for asthma exacerbations, the number of children requiring ED visits or hospitalization for asthma exacerbations, the changes in childhood asthma control test (C-ACT) score, total asthma symptom (TAS) score, forced expiratory volume in the first second (FEV_1_) % predicted, FEV_1_/forced vital capacity (FVC) ratio, forced expiratory flow between 25% and 75% of vital capacity (FEF_25__–75_), fractional exhaled nitric oxide (FeNO), total IgE, and interleukin 10 (IL-10) before and after an intervention. The primary outcome for AD was the change in Scoring Atopic Dermatitis (SCORAD) or the Eczema Area and Severity Index (EASI) before and after an intervention. The secondary outcomes for AD were Staphylococcus aureus skin colonization, the erythema index, and the skin conductance. The primary outcome for AR was symptoms-medication score. The secondary outcome for AR was symptoms score. Other outcomes include changes in serum 25(OH)D concentration before and after an intervention and adverse events. If data were missing or the reporting format was not suitable for the meta-analysis, we contacted the authors of studies by email or calculated from other reported data according to methods recommended by the Cochrane Handbook (Appendix S1 in the Supplementary Materials) [18].

### 2.4. Risk of Bias (RoB) Assessment and Quality of Evidence

Two groups of investigators (group 1 was QL and QZ; group 2 was ZW and YZ) independently assessed the RoB using the Cochrane collaboration risk of bias tool [18]. Each domain was graded as ‘low’, ‘high’, or ‘unclear’ risk. We assessed the quality of the evidence with the Grading of Recommendations Assessment, Development and Evaluation (GRADE) approach for all outcomes [21,22]. GRADE has four levels of evidence: ‘high’, ‘moderate’, ‘low’, or ‘very low’. The quality of evidence from RCTs starts at high and can be downgraded one or two levels due to risk of bias, imprecision, inconsistency, indirectness, and publication bias (e.g., from high to moderate) [21,22]. The disagreement was resolved by a third investigator (YC). Further details are shown in Appendix S1 in the Supplementary Materials.

### 2.5. Data Analysis

Dichotomous outcomes were presented as pooled risk ratios (RRs) and 95% confidence intervals (CIs). Continuous variables were presented as pooled mean differences (MDs) or standard mean differences (SMDs) with 95% CIs. We performed a meta-analysis using the random-effect Mantel-Haenszel model due to diverse population characteristics, intervention, and outcome measures. Heterogeneity was assessed by the *I*^2^ statistic and the value above 50% was suggested substantial statistical heterogeneity [18].

We performed pre-specified subgroup analyses on the following: baseline vitamin D level (serum 25[OH]D < 10 ng/mL vs. 10–19 ng/mL vs. 20–29 ng/mL vs. ≥30 ng/mL), age (1–5 year vs. ≥5 years), the severity of diseases (mild vs. moderate vs. severe), treatment duration (<6 months vs. ≥6 months), a daily dose of vitamin D (daily dose <2000 IU vs. ≥2000 IU), bolus-dose vitamin D given (without bolus dosing vs. administration of at least one bolus dose of vitamin D), and concomitant treatment (use of corticosteroids vs. not). There is currently no consensus definition of an optimal level of vitamin D [23]. Vitamin D levels were typically defined as deficient (serum 25(OH)D < 20 ng/mL), insufficient (20–29 ng/mL), and sufficient (≥30 ng/mL) [24,25]. A cutoff of <10 ng/mL increased the risk of rickets and osteomalacia dramatically, and vitamin D supplementation protected most strongly against acute respiratory infection in patients with baseline 25(OH)D < 10 ng/mL [26,27]. Therefore, serum 25(OH)D < 10 ng/mL was considered to determine severe vitamin D deficiency [23,28]. Based on the above reasons, we selected the 10 ng/mL, 20 ng/mL, and 30 ng/mL cutoffs for baseline serum 25(OH)D concentration in the subgroup analyses.

We also performed a sensitivity analysis to assess the robustness of our findings by excluding trials assessed as having a high risk of bias in one or more of the domains and trials in which mean or SD, or both of them were imputed for missing data. Publication bias was assessed by funnel plot and Egger’s test when there were at least 10 studies included in the meta-analysis. The threshold for significance for p values was 0.05. We performed our data analyses with STATA15.0 (StataCorp, College Station, TX, USA) and RevMan 5.4 software.

## 3. Results

### 3.1. Study Selection and Characteristics

**Figure 1:**
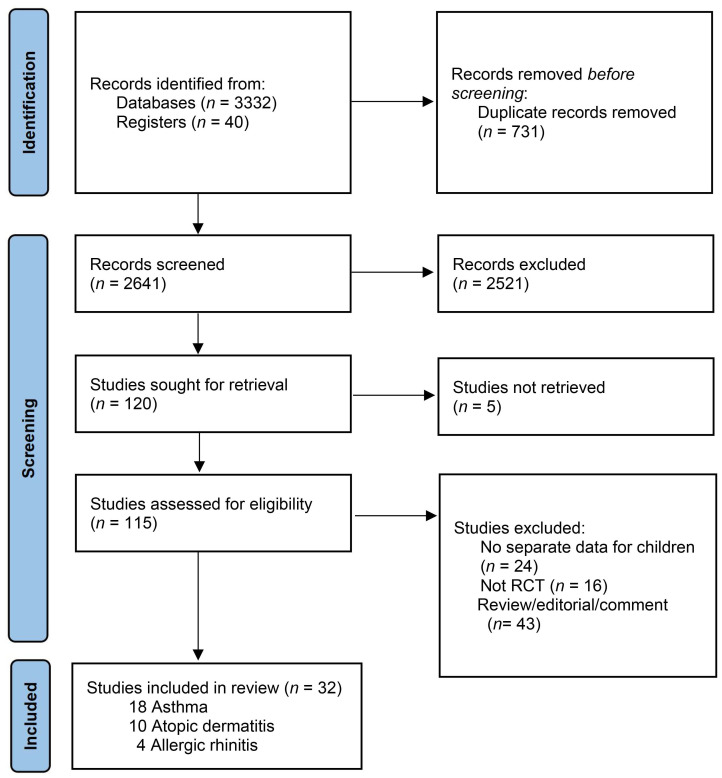
Study flow diagram.

We identified 3372 records with our search strategy, and 115 full-text articles were assessed for eligibility. After excluding 83 articles, 32 RCTs with 2347 participants were included in the review (Figure 1) [29,30,31,32,33,34,35,36,37,38,39,40,41,42,43,44,45,46,47,48,49,50,51,52,53,54,55,56,57,58,59,60]. Among the 32 RCTs, 18 RCTs were for children with asthma [29,30,31,32,33,34,35,36,37,38,39,40,41,42,43,44,45,46], 10 RCTs were for children with AD [47,48,49,50,51,52,53,54,55,56], and 4 RCTs were for children with AR [57,58,59,60]. There were no RCTs for children with food allergies and anaphylaxis. The characteristics of each included RCT are shown in Table 1.

### 3.2. Risk of Bias

Four open-label RCTs did not blind patients and researchers and therefore had high risk of bias in the blinding domain [29,40,50,57]. Five RCTs had high risk of bias in handling missing data [37,46,55,59,60]. Fourteen RCTs had unclear risk of bias in at least one domain [30,31,34,35,36,38,39,41,49,51,53,54,56,58]. Nine RCTs had low risk in all domains [32,33,42,43,44,45,47,48,52]. Details of the RoB assessment results are provided in Figure S1 in the Supplementary Materials.

### 3.3. Childhood Asthma

#### 3.3.1. Overall Analysis

**Figure 2:**
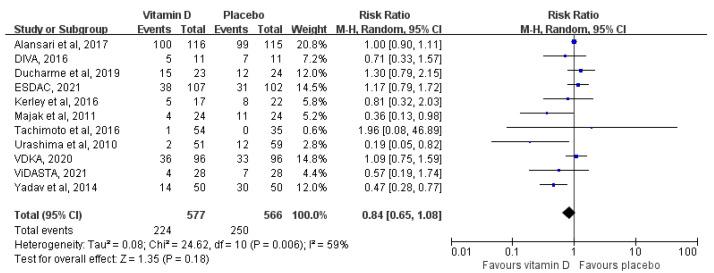
Meta-analysis of the number of children with one or more asthma exacerbations [29,32,33,35,36,38,42,43,44,45,46].

Eleven studies with 1143 patients reported the primary outcome. The pooled estimates found that vitamin D supplementation did not reduce the number of children with one or more asthma exacerbations compared with placebo (RR = 0.84, 95% CI: 0.65 to 1.08, *I*^2^ = 59%, *p* = 0.18) (Figure 2). The quality of this evidence was downgraded to moderate due to their relative imprecision (Table S2 in the Supplementary Materials). Vitamin D supplementation was also not significantly associated with any of the secondary outcomes (Figure S2 in the Supplementary Materials).

#### 3.3.2. Additional Analysis

**Figure 3:**
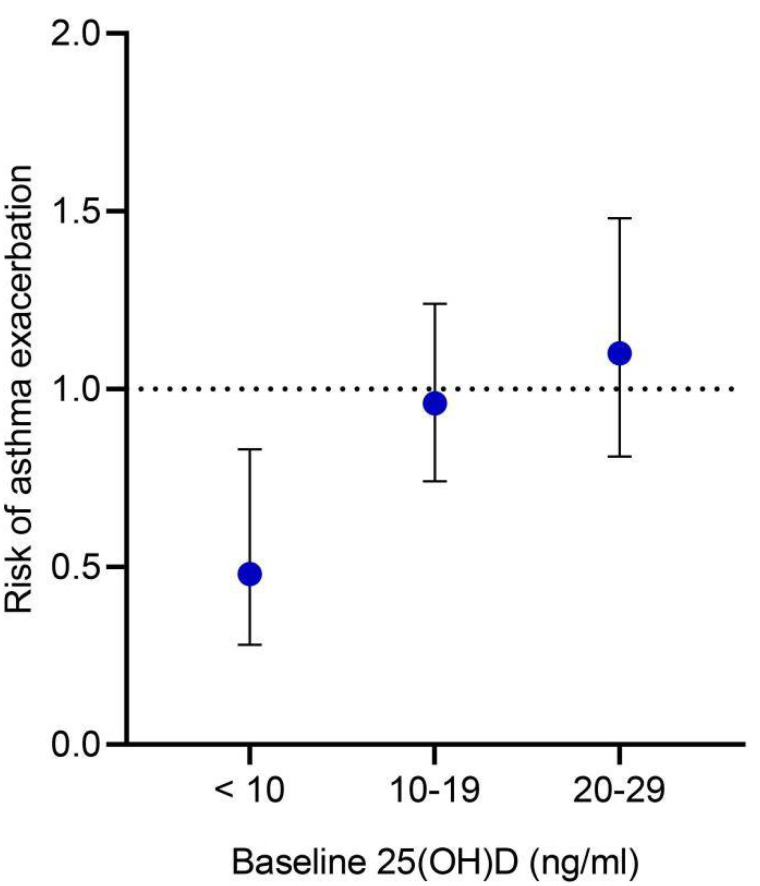
Subgroup analysis of the number of children with one or more asthma exacerbations by baseline serum 25(OH)D concentration. Risk ratio and 95% confidence intervals are presented. Note: we were unable to assess the subgroup of children with baseline 25(OH)D levels ≥ 30 ng/mL because no trials exclusively enrolled those children. As a result, we only examined subgroups with baseline 25(OH)D levels of < 10 ng/mL, 10–19 ng/mL, and 20–29 ng/mL.

We performed subgroup analyses of the primary outcome. We found a statistically significant interaction between asthma exacerbation risk and baseline serum 25(OH)D level (*P_interaction_* = 0.04), indicating that baseline serum 25[OH]D levels modified the effects of vitamin D supplementation on asthma exacerbation risk. The protective effect of vitamin D supplementation on asthma exacerbations decreased with the increase of baseline 25[OH]D levels (Figure 3). Vitamin D supplementation significantly reduced the risk of asthma exacerbation in children with baseline serum 25(OH)D of <10 ng/mL (RR = 0.48, 95% CI: 0.28 to 0.83, *p* = 0.009) (Table S3 in the Supplementary Materials). The quality of this evidence was downgraded to low due to imprecision and a high risk of bias (Table S2 in the Supplementary Materials). However, vitamin D supplementation did not result in a statistically significant reduction in asthma exacerbation risk in children with baseline 25(OH)D between 10 to 19 ng/mL (RR = 0.96, 95% CI: 0.74 to 1.24, *I*^2^ = 9%, *p* = 0.73) or children with baseline 25(OH)D between 20 to 29 ng/mL (RR = 1.10, 95% CI: 0.81 to 1.48, *I*^2^ = 0%, *p* = 0.56) (Table S3 in the Supplementary Materials). We also performed subgroup analyses by age, treatment duration, daily dose of vitamin D, bolus-dose vitamin D given, and concomitant treatment. P values for interaction for these subgroup analyses were higher than 0.05 and no significant protective effects of vitamin D supplementation were found within these subgroups (Table S3 in the Supplementary Materials).

The effect of vitamin D supplementation on the primary outcome remained unchanged when we excluded the trials with a high risk of bias in a sensitivity analysis (RR = 0.88, 95% CI: 0.64 to 1.20, *I*^2^ = 40%, *p* = 0.43) (Figure S3 in the Supplementary Materials). A funnel plot and an Egger’s test (*p* = 0.137) did not observe publication bias in terms of the primary outcome (Figure S4 in the Supplementary Materials).

We also performed subgroup analyses of FEV_1_, FEV_1_/FVC, and total IgE in response to comments from reviewers. Similarly, we did not find protective effects of vitamin D supplementation on these outcomes in each subgroup (Tables S4–S6 in the Supplementary Materials).

### 3.4. Atopic Dermatitis

#### 3.4.1. Overall Analysis

**Figure 4:**
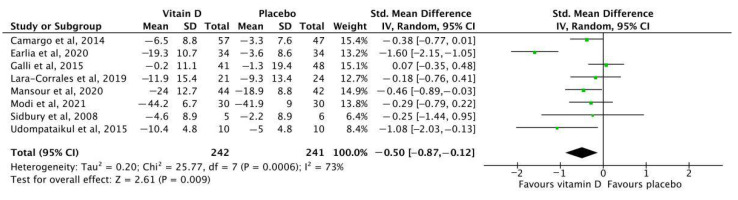
Meta-analysis of the change in EASI or SCORAD before and after intervention among children with atopic dermatitis [48,49,50,51,52,53,54,55]. EASI: Eczema Area and Severity Index; SCORAD: Scoring Atopic Dermatitis.

Eight studies with 483 patients reported the primary outcome. Overall, vitamin D supplementation significantly reduced AD severity assessed by the SCORAD or the EASI score compared with placebo (SMD = −0.5, 95% CI: −0.87 to −0.12, *I*^2^ = 73%, *p* = 0.009) (Figure 4). The quality of this evidence was downgraded to moderate due to inconsistency (Table S2 in the Supplementary Materials). Vitamin D supplementation also improved secondary outcomes. Two studies reported Staphylococcus aureus skin colonization [55,56]. Zulkarnain et al., found that the rate of Staphylococcus aureus skin colonization was significantly lower in the vitamin D supplementation group than in the placebo group [mean (standard deviation, SD): −793.1% (428.5%) vs. −161.5% (787.1%), *p* = 0.043] [56]. Udompataikul et al., found that the vitamin D supplementation group had a lower colony count of Staphylococcus aureus than those taking placebos at week 4 (*p* = 0.08) [55]. They also found that the erythema index of those with vitamin D supplement was statistically significantly lower than those with placebo (*p* = 0.01). Skin conductance was found to be lower in the vitamin D supplementation group, but the difference was not statistically significant (*p* = 0.08).

#### 3.4.2. Additional Analysis

We performed subgroup analyses of the primary outcome. Vitamin D supplementation significantly reduced atopic dermatitis severity in children with baseline serum 25(OH)D of less than 30 ng/mL (SMD = −0.40, 95% CI: −0.67 to −0.13, *I*^2^ = 0%, *p* = 0.004), but not in children with baseline serum 25(OH)D more than or equal to 30 ng/mL (SMD = 0.07, 95% CI: −0.35 to 0.48, *p* = 0.75) (Table S7 in the Supplementary Materials). However, the *p* value for interaction for this subgroup analysis was non-significant (*P_interaction_* = 0.07). The quality of this evidence was downgraded to moderate due to imprecision (Table S2 in the Supplementary Materials). Subgroup analyses by daily dose of vitamin D and bolus-dose vitamin D given did not reveal evidence of effect modification (*P_interaction_* > 0.05) (Table S7 in the Supplementary Materials).

The effect of vitamin D supplementation on the primary outcome remained unchanged when we excluded the trials with a high risk of bias (SMD = −0.55, 95% CI: −0.96 to −0.13, *I*^2^ = 72%, *p* = 0.01) and excluded the trials with missing data (SMD = −0.46, 95% CI: −0.88 to −0.04, *I*^2^ = 79%, *p* = 0.03) in the sensitivity analyses (Figure S5 in the Supplementary Materials).

### 3.5. Allergic Rhinitis

Four studies with 307 patients reported the outcomes of children with allergic rhinitis. Data from these studies could not be pooled because of high heterogeneity in the outcome reporting. Therefore, we performed a qualitative analysis. Jerzynska’s study in 2016 found that when compared with placebo group, the vitamin D group was more effective in the reduction of symptom-medication score (mean (SD): 43.7 (3.3) vs. 57.8 (4.4), *p* = 0.001), nasal symptoms (mean (SD): 17.3 (1.8) vs. 22.4 (2.5), *p* = 0.04), and asthma symptoms (mean (SD): 7.7 (1.0) vs. 12.8 (1.7), *p* = 0.001). No significant difference was found between the groups in medication (*p* = 0.211) and ocular scores (*p* = 0.149) [59]. Jerzynska’s study in 2018 found that the symptom-medication score was significantly lower in vitamin D supplementation group compared to placebo group (*p* = 0.0371) [60]. Akram et al. found that children with AR had a significant lower symptom score of rhinorrhea (*p* < 0.001), nasal congestion (*p* = 0.01), sneezing (*p* < 0.001), and itching (*p* = 0.016) in the vitamin D supplementation group than in the placebo group [57]. Hassan et al. found that improvement in the average morning and evening total nasal symptom scores was significantly greater in vitamin D supplementation group compared to placebo group (improved mean score: −0.61, 95% CI: −1.05 to −0.18) [58].

### 3.6. Serum 25(OH)D Concentration Levels

Twenty-one RCTs (n = 1545) reported the changes of serum 25(OH)D levels before and after intervention. The increase of serum 25(OH)D in children with vitamin D supplementation was significantly greater than that in the control group (MD = 16.84, 95% CI: 11.75 to 21.93, *p <* 0.001) (Figure S6 in the Supplementary Materials). However, after vitamin D supplementation, the mean serum 25(OH)D level of children with asthma was only 33.48 (95% CI: 27.94 to 39.02) ng/mL. The mean post-intervention serum 25(OH)D levels in children with atopic dermatitis and allergic rhinitis were 67.69 (95% CI: 47.19 to 88.19) ng/mL and 75.42 (95% CI: 30.90 to 119.93) ng/mL, respectively (Figure S7 in the Supplementary Materials).

### 3.7. Adverse Events

Two RCTs giving a bolus dose of 100,000 IU vitamin D reported that eight children had serum 25(OH)D levels greater than 90 ng/mL after taking vitamin D [32,33]. However, urinary Ca:Cr was normal in these children [32,33]. In one study, one child in the vitamin D group and one in the placebo group developed hypercalciuria [32]. No participant had hypercalcemia, renal stones, or suppressed parathyroid hormone. Vitamin D supplementation did not increase the risk of any non-serious or serious adverse events (Table S8 in the Supplementary Materials).

## 4. Discussion

In this meta-analysis, vitamin D supplementation did not reduce the risk of asthma exacerbation and did not improve asthma control and lung function in children overall but may reduce asthma exacerbation risk in children with serum 25(OH)D concentration less than 10 ng/mL. We also found that vitamin D supplementation can reduce the severity of AD in children, especially children with serum 25(OH)D concentration less than 30 ng/mL. In addition, vitamin D supplementation has been shown to significantly alleviate symptoms in children with allergic rhinitis. Vitamin D supplementation was safe and did not increase the risk of severe adverse events.

Our findings are consistent with those of a recently published meta-analysis, which did not find protective effects of vitamin D supplementation on reducing asthma exacerbation in children either [13]. There are many factors that contribute to the ineffectiveness of vitamin D supplementation in children with asthma. First and foremost, vitamin D supplementation may be effective in particular subgroups, rather than all children. Our subgroup analysis found a protective effect of vitamin D on asthmatic children with baseline 25(OH)D of less than 10 ng/mL, but not in patients with higher baseline 25(OH)D levels. A meta-analysis of individual participant data yielded the same results [11]. Although both meta-analyses found that vitamin D supplementation was effective in asthmatic patients with severe vitamin D deficiency, the sample size of this subgroup was small. More large-sample RCTs are required in the future to confirm this finding. In addition, the influence of vitamin D on childhood asthma may be affected by genetic differences in vitamin D metabolic pathways [61]. Some studies suggested that vitamin D may be more beneficial in severe steroid-resistant asthma and asthmatic children with obesity [62,63]. However, the influence of vitamin D supplementation in these subgroups could not be evaluated in our meta-analysis due to the unavailability of data. Additional large, rigorously designed RCTs in these fields are needed. A second reason may be inadequate vitamin D supplementation. Serum 25(OH)D concentrations more than 30 ng/mL are widely accepted to indicate vitamin D sufficiency [64]. However, this standard is suitable for bone health [64]. Some children with asthma still had recurrent attacks with serum 25(OH)D more than 30 ng/mL [65]. Thus, some experts suggest the optimum serum 25(OH)D level for allergic and inflammatory diseases should be above 40 ng/mL [24,66]. In our meta-analysis, after vitamin D supplementation, the mean serum 25(OH)D level of children with asthma was only 33.48 (95% CI: 27.94 to 39.02) ng/mL, which indicated that nearly half of the patients did not achieve serum 25(OH)D level of 30 ng/mL after vitamin D supplementation, and more than 95% of the patients did not achieve serum 25(OH)D level of 40 ng/mL. In contrast to RCTs on AD and AR, the pooled results of these RCTs showed vitamin D supplementation was effective, and accordingly, the mean serum 25(OH)D levels after vitamin D supplementation in these study populations were much higher than those in children with asthma and also higher than 40 ng/mL. Future RCTs should explore the effectiveness of vitamin D in children with asthma after adequate levels of 25(OH)D have been reached.

Consistent with our findings, vitamin D supplementation has shown to reduce the severity of AD in children in other meta-analyses [14,67,68,69]. However, the three previously published meta-analyses included both pediatric and adult patients [24,67,68]. A recently published meta-analysis only analyzed the results of four trials in pediatric patients and did not conduct subgroup analyses [14]. In our subgroup analysis, we saw a statistically significant reduction in AD severity with baseline 25(OH)D of less than or equal to 30 ng/mL, but not in participants with 25(OH)D of more than 30 ng/mL. However, the p value for interaction for this subgroup analysis was non-significant. Formally, we have not shown that effects are stronger in one group than in the other; alternatively, we might have lacked statistical power to detect the relevant interactions. Further research to clarify whether baseline vitamin D status modifies the effects of vitamin D on AD severity is needed.

There has been no SR evaluating the efficacy of vitamin D supplementation in children with AR. Our SR included four RCTs on children with AR and the results of each RCT showed that vitamin D supplementation significantly reduced the symptoms of AR patients [57,58,59,60]. However, some RCTs had design issues, such as not blinding participants and researchers and not properly addressing missing data [57,59,60]. In addition, we were unable to perform a quantitative analysis because of differences in outcomes between trials. Therefore, in the future, researchers need to design their trials in accordance with the Cochrane Risk-of-bias Tool for Randomized Trials [18] and investigate standardized and patient-relevant outcomes that are comparable across studies.

There has been no RCTs evaluating the efficacy of vitamin D supplementation in children with food allergies and anaphylaxis. Future research on these allergic diseases may be needed. P values for interaction were higher than 0.05 for subgroup analyses relating to vitamin D dosing regimen, treatment duration, and use of corticosteroids. Therefore, we did not find evidence that effects of vitamin D supplementation differed across these subgroups of patients. As a result, the optimum dose and treatment duration of vitamin D and the synergistic effects of vitamin D and corticosteroids are as yet unknown and require additional primary studies to determine.

We provided a comprehensive analysis of the effects of vitamin D supplementation on allergic diseases in children and compared the effects across allergic diseases and populations for the first time. However, we acknowledge several limitations of our study. First, there was variability in the population characteristics, intervention, and outcomes, which may lead to heterogeneity in results. Therefore, we pooled the data using a random-effects model. In addition, we performed subgroup analyses and sensitivity analyses and found the results remained unchanged and statistical heterogeneity was reduced. Second, we could not conduct some of the prespecified subgroup analyses because relevant information was not reported in the primary studies. Individual patient data meta-analysis of existing datasets is needed before definitive clinical recommendations can be made.

## 5. Conclusions

Vitamin D supplementation did not reduce the risk of asthma exacerbations in children overall but may reduce asthma exacerbation risk in children with serum 25(OH)D concentration less than 10 ng/mL. Vitamin D supplementation can safely reduce the severity of AD and symptoms of AR in children. Large-scale and well-designed RCTs are needed to confirm these conclusions and investigate the optimum regimen of vitamin D and the patients who would benefit most from vitamin D supplementation.

## Figures and Tables

**Table 1:** Study characteristics.

First Author (year)	Sample Size	Country	Disease	Disease Severity	Age, Years, Range	Male (%)	BMI, kg/m^2^, Mean (SD)	Baseline 25(OH)D, ng/mL, Mean (SD)	Co-Treatment	Dose of Vitamin D	Treatment Duration	Primary Outcome
Alansari et al. (2017) [29]	231	USA	Asthma	Moderate to severe	2–14	64.9	NR	15.4 (5.3)	None	300,000–600,000 IU, statim followed by 400 IU daily	12 months	Unplanned visit for asthma exacerbation
Bar Yoseph et al. (2015) [30]	39	Israel	Asthma	Mild	6–18	64.1	20.6 (3.7)	20.4 (6.7)	None	14000 IU weekly	6 weeks	Lung functions
Baris et al. (2014) [31]	32	Turkey	Asthma	Mild to moderate	5–15	37.5	NR	19.5 (10.3)	SCIT	650 IU daily	12 months	Asthma control assessed by TASS
DIVA (2016) [32]	22	Canada	Asthma	All severity	1–5	31.8	NR	25.3 (8.2)	None	100,000 IU statim followed by 400 IU daily	6 months	Incidence of asthma exacerbation
Ducharme et al. (2019) [33]	47	Canada	Asthma	All severity	1–5	63.8	NR	NR	ICS	100,000 IU × 2 doses, 14 weeks apart	7 months	Incidence of asthma exacerbation
EI-Korashi et al. (2021) [34]	46	Egypt	Asthma	Mild to moderate	1–18	52.2	NR	13.0 (4.0)	SCIT	600 IU daily	6 months	Change in serum level of IL-10
ESDAC (2021) [35]	250	India	Asthma	All severity	4–12	72	NR	11.2 (4.5)	None	1000 IU daily	9 months	Asthma control assessed by C-ACT
Kerley et al. (2016) [36]	39	Ireland	Asthma	All severity	6–16	61.5	18.8 (3.7)	21.2 (8.7)	None	2000 IU daily	15 weeks	Lung functions
Lewis et al. (2012) [37]	30	USA	Asthma	All severity	6–17	NR	NR	NR	None	1000 IU daily	12 months	Asthma control assessed by ACT
Majak et al. (2009) [38]	36	Poland	Asthma	All severity	6–12	61.1	NR	31.7 (3.2)	ICS + SCIT	1000 IU weekly	3 months	ICS dose reduction
Majak et al. (2011) [39]	48	Poland	Asthma	All severity	5–18	66.7	18.7 (4.1)	35.6 (15.3)	ICS	500 IU daily	6 months	Incidence of asthma exacerbation
Najmuddin et al. (2017) [40]	66	India	Asthma	All severity	6–12	63.6	NR	NR	None	60,000 IU weekly	10 weeks	Lung functions
Swangtrakul et al. (2022) [41]	41	Thailand	Asthma	All severity	3–18	48.8	19.7 (4.3)	16.4 (2.2)	None	<30 kg: 300,000 IU; >30 kg: 600,000 IU	3 months	Lung functions
Tachimoto et al. (2016) [42]	89	Japan	Asthma	All severity	6–15	56.2	17.5 (2.7)	29.4 (6.8)	None	800 IU daily	2 months	Asthma control assessed by GINA
Urashima et al. (2010) [43]	110	Japan	Asthma	All severity	6–15	56.3	NR	NR	None	1200 IU daily	4 months	Incidence of asthma exacerbation
VDKA (2020) [44]	192	USA	Asthma	Mild to moderate	6–16	59.9	NR	22.7 (4.6)	ICS	4000 IU daily	12 months	Time to a severe asthma exacerbation
ViDASTA (2021) [45]	60	India	Asthma	Moderate	6–11	56.7	NR	16.2 (9.0)	ICS	2000 IU daily	3 months	Asthma control assessed by C-ACT
Yadav et al. (2014) [46]	100	India	Asthma	Moderate to severe	5–13	49	NR	NR	None	60,000 IU monthly	6 months	Incidence of asthma exacerbation
Aldaghi et al. (2021) [47]	54	Iran	Atopic dermatitis	All severity	0–1	51.9	NR	NR	Corticosteroids, emollient, and antihistamines	1000 IU daily	2 months	Disease severity assessed by SCORAD
Camargo et al. (2014) [48]	107	Mongolia	Atopic dermatitis	All severity	2–17	58.9	NR	NR	Emollient, patient education, and basic skin care	1000 IU daily	1 month	Disease severity assessed by EASI
Earlia et al. (2020) [49]	68	Indonesia	Atopic dermatitis	All severity	1–18	50	NR	NR	Corticosteroids, emollient, and antihistamines	600 IU daily	28 days	Disease severity assessed by SCORAD
Galli et al. (2015) [50]	89	Italy	Atopic dermatitis	All severity	0.5–16.25	53.9	NR	48.3 (40.6)	None	2000 IU daily	3 months	Disease severity assessed by SCORAD score
Lara-Corrales et al. (2019) [51]	45	Canada	Atopic dermatitis	All severity	0–18	53.3	NR	17.8 (6.2)	None	1000 IU daily	3 months	Disease severity assessed by SCORAD
Mansour et al. (2020) [52]	92	Egypt	Atopic dermatitis	All severity	5–16	51.2	26.9 (5.0)	24.1 (7.3)	Corticosteroids	1600 IU daily	3 months	Disease severity assessed by EASI
Modi et al. (2021) [53]	60	India	Atopic dermatitis	Moderate to severe	1–14	55	NR	17.5 (2.8)	Regular treatment	60,000 IU weekly	6 weeks	Disease severity assessed by SCORAD
Sidbury et al. (2008) [54]	11	USA	Atopic dermatitis	Mild	2–13	54.5	NR	NR	Previously prescribed therapies	1000 IU daily	1 month	Disease severity assessed by EASI
Udompataikul et al. (2015) [55]	20	Thailand	Atopic dermatitis	Mild to moderate	1–18	35	NR	17.0 (1.6)	Antihistamine, sunscreen, skin moisturizer, and cleanser	2000 IU daily	1 month	Disease severity assessed by SCORAD
Zulkarnain et al. (2019) [56]	20	Indonesia	Atopic dermatitis	All severity	2–12	60	NR	NR	None	400 IU daily	28 days	Staphylococcus aureus colonization
Akram et al. (2020) [57]	120	Pakistan	Allergic rhinitis	Moderate to severe	5–15	53.3	NR	23.3 (11.9)	Standard treatment	800 IU daily	1 month	Symptom score
Hassan et al. (2016) [58]	100	Egypt	Allergic rhinitis	All severity	6–12	50	27.9 (4.9)	18.4 (6.1)	None	1000 IU daily	6 months	Symptoms score
Jerzynska et al. (2016) [59]	45	Poland	Allergic rhinitis	Moderate to severe	5–12	57.8	NR	45.9 (5.2)	SLIT and standard treatment	1000 IU daily	5 months	Symptom-medication score
Jerzynska et al. (2018) [60]	38	Poland	Allergic rhinitis	Moderate to severe	5–12	NR	NR	60.9 (36.7)	Standard treatment	1000 IU daily	5 months	Symptom-medication score

Abbreviation: BMI: body mass index; C-ACT: childhood asthma control test; EASI: eczema area and severity index; GINA: global initiative for asthma; ICS: inhaled corticosteroids; NR: not reported; SCIT: subcutaneous immunotherapy; SCORAD: scoring atopic dermatitis; SD: standard deviation; TASS: total asthma symptom score.

## Data Availability

Not applicable.

## Supplementary Materials

The following supporting information can be downloaded online https://www.mdpi.com/article/10.3390/nu14193947/s1. Table S1: Search strategies, Table S2: GRADE assessment (Summary of Findings table), Table S3: Subgroup analyses of number of children with one or more asthma exacerbations, Table S4: Subgroup analyses of change in forced expiratory volume in the first second (FEV_1_) % predicted, Table S5: Subgroup analyses of change in FEV_1_/forced vital capacity (FVC) ratio; Table S6: Subgroup analyses of change in total IgE, Table S7: Subgroup analyses of atopic dermatitis severity, Table S8: Adverse events reported in the included RCTs, Figure S1: Assessment on risk of bias for included RCTs, Figure S2: Meta analyses of secondary outcomes among children with asthma, Figure S3: Sensitivity analyses of number of children with one or more asthma exacerbations, Figure S4: Publication bias of number of children with one or more asthma exacerbations, Figure S5: Sensitivity analyses of atopic dermatitis severity, Figure S6: Meta analyses of change in serum 25(OH)D concentration before and after intervention, Figure S7: Meta analyses of post-intervention serum 25(OH)D concentration in vitamin D group, Appendix S1: Methods [18,21,22].

## References

[1] Dierick B.J.H., van der Molen T., Flokstra-de Blok B.M.J., Muraro A., Postma M.J., Kocks J.W.H., van Boven J.F.M. (2020). Burden and socioeconomics of asthma, allergic rhinitis, atopic dermatitis and food allergy. Expert Rev. Pharm. Outcomes Res..

[2] Pawankar R. (2014). Allergic diseases and asthma: A global public health concern and a call to action. World Allergy Organ. J..

[3] Mirzakhani H., Al-Garawi A., Weiss S.T., Litonjua A.A. (2015). Vitamin D and the development of allergic disease: How important is it?. Clin. Exp. Allergy.

[4] Schrumpf J.A., van der Does A.M., Hiemstra P.S. (2020). Impact of the Local Inflammatory Environment on Mucosal Vitamin D Metabolism and Signaling in Chronic Inflammatory Lung Diseases. Front. Immunol..

[5] Vasiliou J.E., Lui S., Walker S.A., Chohan V., Xystrakis E., Bush A., Hawrylowicz C.M., Saglani S., Lloyd C.M. (2014). Vitamin D deficiency induces Th2 skewing and eosinophilia in neonatal allergic airways disease. Allergy.

[6] Rolf L., Muris A.H., Hupperts R., Damoiseaux J. (2014). Vitamin D effects on B cell function in autoimmunity. Ann. N. Y. Acad. Sci..

[7] Kuruvilla M.E., Lee F.E., Lee G.B. (2019). Understanding Asthma Phenotypes, Endotypes, and Mechanisms of Disease. Clin. Rev. Allergy Immunol..

[8] Han Y.Y., Forno E., Celedón J.C. (2017). Vitamin D Insufficiency and Asthma in a US Nationwide Study. J. Allergy Clin. Immunol. Pract..

[9] Bunyavanich S., Rifas-Shiman S.L., Platts-Mills T.A., Workman L., Sordillo J.E., Camargo C.A., Gillman M.W., Gold D.R., Litonjua A.A. (2016). Prenatal, perinatal, and childhood vitamin D exposure and their association with childhood allergic rhinitis and allergic sensitization. J. Allergy Clin. Immunol..

[10] Ahmed Mohamed A., Salah Ahmed E.M., Farag Y.M.K., Bedair N.I., Nassar N.A., Ghanem A.I.M. (2021). Dose-response association between vitamin D deficiency and atopic dermatitis in children, and effect modification by gender: A case-control study. J. Dermatolog. Treat..

[11] Jolliffe D.A., Greenberg L., Hooper R.L., Griffiths C.J., Camargo C.A., Kerley C.P., Jensen M.E., Mauger D., Stelmach I., Urashima M. (2017). Vitamin D supplementation to prevent asthma exacerbations: A systematic review and meta-analysis of individual participant data. Lancet Respir. Med..

[12] Martineau A.R., Cates C.J., Urashima M., Jensen M., Griffiths A.P., Nurmatov U., Sheikh A., Griffiths C.J. (2016). Vitamin D for the management of asthma. Cochrane Database Syst. Rev..

[13] Kumar J., Kumar P., Goyal J.P., Thakur C., Choudhary P., Meena J., Charan J., Singh K., Gupta A. (2021). Vitamin D supplementation in childhood asthma: A systematic review and meta-analysis of randomised controlled trials. ERJ Open Res..

[14] Hidayati A.N., Sawitri S., Sari D.W., Prakoeswa C.R.S., Indramaya D.M., Damayanti D., Zulkarnain I., Citrashanty I., Widia Y., Anggraeni S. (2022). Efficacy of vitamin D supplementation on the severity of atopic dermatitis in children: A systematic review and meta-analysis [version 1; peer review: 1 approved]. F1000Research.

[15] Tran M.M., Lefebvre D.L., Dharma C., Dai D., Lou W.Y.W., Subbarao P., Becker A.B., Mandhane P.J., Turvey S.E., Sears M.R. (2018). Predicting the atopic march: Results from the Canadian Healthy Infant Longitudinal Development Study. J. Allergy Clin. Immunol..

[16] Giovannini-Chami L., Paquet A., Sanfiorenzo C., Pons N., Cazareth J., Magnone V., Lebrigand K., Chevalier B., Vallauri A., Julia V. (2018). The “one airway, one disease” concept in light of Th2 inflammation. Eur. Respir. J..

[17] Lenaeus M.J., Hirschmann J. (2015). Primary Care of the Patient with Asthma. Med. Clin. N. Am..

[18] Higgins J.P.T., Thomas J., Chandler J., Cumpston M., Li T., Page M.J., Welch V.A. (2019). Cochrane Handbook for Systematic Reviews of Interventions.

[19] Page M.J., McKenzie J.E., Bossuyt P.M., Boutron I., Hoffmann T.C., Mulrow C.D., Shamseer L., Tetzlaff J.M., Akl E.A., Brennan S.E. (2021). The PRISMA 2020 statement: An updated guideline for reporting systematic reviews. BMJ.

[20] Davidson B.L. (2018). Administration of placebo vitamin D to non-consenting children. Lancet Respir. Med..

[21] Guyatt G.H., Oxman A.D., Vist G.E., Kunz R., Falck-Ytter Y., Alonso-Coello P., Schünemann H.J., GRADE Working Group (2008). GRADE: An emerging consensus on rating quality of evidence and strength of recommendations. BMJ.

[22] Guyatt G.H., Oxman A.D., Santesso N., Helfand M., Vist G., Kunz R., Brozek J., Norris S., Meerpohl J., Djulbegovic B. (2013). GRADE guidelines: 12. Preparing summary of findings tables-binary outcomes. J. Clin. Epidemiol..

[23] Amrein K., Scherkl M., Hoffmann M., Neuwersch-Sommeregger S., Köstenberger M., Tmava Berisha A., Martucci G., Pilz S., Malle O. (2020). Vitamin D deficiency 2.0, an update on the current status worldwide. Eur. J. Clin. Nutr..

[24] Holick M.F., Binkley N.C., Bischoff-Ferrari H.A., Gordon C.M., Hanley D.A., Heaney R.P., Murad M.H., Weaver C.M., Endocrine Society (2011). Evaluation, treatment, and prevention of vitamin D deficiency: An Endocrine Society Clinical Practice Guideline. J. Clin. Endocrinol. Metab..

[25] Institute of Medicine (2011). Dietary Reference Intakes for Calcium and Vitamin D.

[26] Cashman K.D., Fitzgerald A.P., Kiely M., Seamans K.M. (2011). A systematic review and meta-regression analysis of the vitamin D intake-serum 25-hydroxyvitamin D relationship to inform European recommendations. Br. J. Nutr..

[27] Martineau A.R., Jolliffe D.A., Hooper R.L., Greenberg L., Aloia J.F., Bergman P., Dubnov-Raz G., Esposito S., Ganmaa D., Ginde A.A. (2017). Vitamin D supplementation to prevent acute respiratory tract infections: Systematic review and meta-analysis of individual participant data. BMJ.

[28] Braegger C., Campoy C., Colomb V., Decsi T., Domellof M., Fewtrell M., Hojsak I., Mihatsch W., Molgaard C., Shamir R. (2013). Vitamin D in the healthy European paediatric population. J. Pediatr. Gastroenterol. Nutr..

[29] Alansari K., Davidson B.L., Yousef K.I., Mohamed A.N.H., Alattar I. (2017). Rapid vs. Maintenance Vitamin D Supplementation in Deficient Children With Asthma to Prevent Exacerbations. Chest.

[30] Bar Yoseph R., Livnat G., Schnapp Z., Hakim F., Dabbah H., Goldbart A., Bentur L. (2015). The effect of vitamin D on airway reactivity and inflammation in asthmatic children: A double-blind placebo-controlled trial. Pediatr. Pulmonol..

[31] Baris S., Kiykim A., Ozen A., Tulunay A., Karakoc-Aydiner E., Barlan I.B. (2014). Vitamin D as an adjunct to subcutaneous allergen immunotherapy in asthmatic children sensitized to house dust mite. Allergy.

[32] Jensen M.E., Mailhot G., Alos N., Rousseau E., White J.H., Khamessan A., Ducharme F.M. (2016). Vitamin D intervention in preschoolers with viral-induced asthma (DIVA): A pilot randomised controlled trial. Trials.

[33] Ducharme F.M., Jensen M., Mailhot G., Alos N., White J., Rousseau E., Tse S.M., Khamessan A., Vinet B. (2019). Impact of two oral doses of 100,000 IU of vitamin D3 in preschoolers with viral-induced asthma: A pilot randomised controlled trial. Trials.

[34] El-korashi L.A., Nafea O.E., Zake L.G., Arab F., Anis R.H. (2021). Effect of Vitamin D Adjuvant and Allergen Specific Immunotherapy on Serum IL-10 and IL-17 Levels in Childhood Asthma: A Controlled Clinical Trial. Egypt J. Med. Microbiol..

[35] Jat K.R., Goel N., Gupta N., Gupta C.P., Datta S., Lodha R., Kabra S.K. (2021). Efficacy of vitamin D supplementation in asthmatic children with vitamin D deficiency: A randomized controlled trial (ESDAC trial). Pediatr. Allergy Immunol..

[36] Kerley C.P., Hutchinson K., Cormican L., Faul J., Greally P., Coghlan D., Elnazir B. (2016). Vitamin D3 for uncontrolled childhood asthma: A pilot study. Pediatr. Allergy Immunol..

[37] Lewis E., Fernandez C., Nella A., Hopp R., Gallagher J.C., Casale T.B. (2012). Relationship of 25-hydroxyvitamin D and asthma control in children. Ann. Allergy Asthma Immunol..

[38] Majak P., Rychlik B., Stelmach I. (2009). The effect of oral steroids with and without vitamin D3 on early efficacy of immunotherapy in asthmatic children. Clin. Exp. Allergy.

[39] Majak P., Olszowiec-Chlebna M., Smejda K., Stelmach I. (2011). Vitamin D supplementation in children may prevent asthma exacerbation triggered by acute respiratory infection. J. Allergy Clin. Immunol..

[40] Najmuddin F., Lahiri K. (2017). Vitamin D in pediatric asthma and allergic rhinitis: Benefits beyond skeletal health. Insights Allergy Asthma Bronchitis.

[41] Swangtrakul N., Manuyakorn W., Mahachoklertwattana P., Kiewngam P., Sasisakulporn C., Jotikasthirapa W., Kamchaisatian W., Benjaponpitak S. (2022). Effect of vitamin D on lung function assessed by forced oscillation technique in asthmatic children with vitamin D deficiency: A randomized double-blind placebo-controlled trial. Asian Pac. J. Allergy Immunol..

[42] Tachimoto H., Mezawa H., Segawa T., Akiyama N., Ida H., Urashima M. (2016). Improved control of childhood asthma with low-dose, short-term vitamin D supplementation: A randomized, double-blind, placebo-controlled trial. Allergy.

[43] Urashima M., Segawa T., Okazaki M., Kurihara M., Wada Y., Ida H. (2010). Randomized trial of vitamin D supplementation to prevent seasonal influenza A in schoolchildren. Am. J. Clin. Nutr..

[44] Forno E., Bacharier L.B., Phipatanakul W., Guilbert T.W., Cabana M.D., Ross K., Covar R., Gern J.E., Rosser F.J., Blatter J. (2020). Effect of Vitamin D3 Supplementation on Severe Asthma Exacerbations in Children With Asthma and Low Vitamin D Levels: The VDKA Randomized Clinical Trial. JAMA.

[45] Thakur C., Kumar J., Kumar P., Goyal J.P., Singh K., Gupta A. (2021). Vitamin-D supplementation as an adjunct to standard treatment of asthma in children: A randomized controlled trial (ViDASTA Trial). Pediatr. Pulmonol..

[46] Yadav M., Mittal K. (2014). Effect of vitamin D supplementation on moderate to severe bronchial asthma. Indian J. Pediatr..

[47] Aldaghi M., Tehrani H., Karrabi M., Abadi F.S., Sahebkar M. (2022). The effect of multistrain synbiotic and vitamin D3 supplements on the severity of atopic dermatitis among infants under 1 year of age: A double-blind, randomized clinical trial study. J. Dermatol. Treat..

[48] Camargo C.A., Ganmaa D., Sidbury R., Erdenedelger Kh Radnaakhand N., Khandsuren B. (2014). Randomized trial of vitamin D supplementation for winter-related atopic dermatitis in children. J. Allergy Clin. Immunol..

[49] Earlia N., Maulida M., Hidayati A., Pratama R. (2020). Pengaruh Pemberian Vitamin D Terhadap Perbaikan Gejala Klinis Pada Penderita Dermatitis Atopik Di Poliklinik Kulit Kelamin RSUD Dr. Zainoel Abidin Banda Aceh Tahun 2018: Uji Klinis Ketersamaran Ganda. J. Med. Sci..

[50] Galli E., Rocchi L., Carello R., Giampietro P.G., Panei P., Meglio P. (2015). Serum Vitamin D levels and Vitamin D supplementation do not correlate with the severity of chronic eczema in children. Eur. Ann. Allergy Clin. Immunol..

[51] Lara-Corrales I., Huang C.M., Parkin P.C., Rubio-Gomez G.A., Posso-De Los Rios C.J., Maguire J., Pope E. (2019). Vitamin D Level and Supplementation in Pediatric Atopic Dermatitis: A Randomized Controlled Trial. J. Cutan. Med. Surg..

[52] Mansour N.O., Mohamed A.A., Hussein M., Eldemiry E., Daifalla A., Hassanin S., Nassar N., Ghaith D., Mohamed Salah E. (2020). The impact of vitamin D supplementation as an adjuvant therapy on clinical outcomes in patients with severe atopic dermatitis: A randomized controlled trial. Pharmacol. Res. Perspect..

[53] Modi N.P., Dash A.K. (2021). Clinico-biochemical relation of Vitamin D3 with the severity of atopic dermatitis and response to supplementation of Vitamin D3: A randomized controlled trial. Ind. J. Child. Health.

[54] Sidbury R., Sullivan A.F., Thadhani R.I., Camargo C.A. (2008). Randomized controlled trial of vitamin D supplementation for winter-related atopic dermatitis in Boston: A pilot study. Br. J. Dermatol..

[55] Udompataikul M., Huajai S., Chalermchai T., Taweechotipatr M., Kamanamool N. (2015). The Effects of Oral Vitamin D Supplement on Atopic Dermatitis: A Clinical Trial with Staphylococcus aureus Colonization Determination. J. Med. Assoc. Thail..

[56] Zulkarnain I., Rahmawati Y.W., Setyaningrum T., Citrashanty I., Aditama L., Avanti C. (2019). Vitamin D3 supplementation reduced Staphylococcus aureus colonization in the skin of pediatric patients with atopic dermatitis. Eur. J. Pediatr. Dermatol..

[57] Akram S., Khan M.A., Fazil M., Kiramatullah Khan M.Q., Shah H.B.U. (2020). Effect of Vitamin-D Supplementation in Children with Moderate-Severe Persistent Allergic Rhinitis. Int. J. Pathol..

[58] Hassan S.M., Elmageed M.M.A., Senosy M.G.E. (2016). Vitamin D Intervention in Children with Allergic rhinitis: A Pilot Randomized Controlled Trial. Int. J. Curr. Microbiol. Appl. Sci..

[59] Jerzynska J., Stelmach W., Rychlik B., Lechańska J., Podlecka D., Stelmach I. (2016). The clinical effect of vitamin D supplementation combined with grass-specific sublingual immunotherapy in children with allergic rhinitis. Allergy Asthma Proc..

[60] Jerzyńska J., Stelmach W., Rychlik B., Majak P., Podlecka D., Woicka-Kolejwa K., Stelmach I. (2018). Clinical and immunological effects of vitamin D supplementation during the pollen season in children with allergic rhinitis. Arch. Med. Sci..

[61] Zhao D.D., Yu D.D., Ren Q.Q., Dong B., Zhao F., Sun Y.H. (2017). Association of vitamin D receptor gene polymorphisms with susceptibility to childhood asthma: A meta-analysis. Pediatr. Pulmonol..

[62] Nanzer A.M., Chambers E.S., Ryanna K., Freeman A.T., Colligan G., Richards D.F., Timms P.M., Martineau A.R., Griffiths C.J., Corrigan C.J. (2014). The effects of calcitriol treatment in glucocorticoid-resistant asthma. J. Allergy Clin. Immunol..

[63] Bose S., Diette G.B., Woo H., Koehler K., Romero K., Rule A.M., Detrick B., Brigham E., McCormack M.C., Hansel N.N. (2019). Vitamin D Status Modifies the Response to Indoor Particulate Matter in Obese Urban Children with Asthma. J. Allergy Clin. Immunol. Pract..

[64] Ross A.C., Manson J.E., Abrams S.A., Aloia J.F., Brannon P.M., Clinton S.K., Durazo-Arvizu R.A., Gallagher J.C., Gallo R.L., Jones G. (2011). The 2011 report on dietary reference intakes for calcium and vitamin D from the Institute of Medicine: What clinicians need to know. J. Clin. Endocrinol. Metab..

[65] Luo C., Sun Y., Zeng Z., Liu Y., Peng S. (2022). Vitamin D supplementation in pregnant women or infants for preventing allergic diseases: A systematic review and meta-analysis of randomized controlled trials. Chin. Med. J..

[66] Hollis B.W., Wagner C.L., Drezner M.K., Binkley N.C. (2007). Circulating vitamin D3 and 25-hydroxyvitamin D in humans: An important tool to define adequate nutritional vitamin D status. J. Steroid Biochem. Mol. Biol..

[67] Hattangdi-Haridas S.R., Lanham-New S.A., Wong W.H.S., Ho M.H.K., Darling A.L. (2019). Vitamin D Deficiency and Effects of Vitamin D Supplementation on Disease Severity in Patients with Atopic Dermatitis: A Systematic Review and Meta-Analysis in Adults and Children. Nutrients.

[68] Kim M.J., Kim S.N., Lee Y.W., Choe Y.B., Ahn K.J. (2016). Vitamin D Status and Efficacy of Vitamin D Supplementation in Atopic Dermatitis: A Systematic Review and Meta-Analysis. Nutrients.

[69] Kim G., Bae J.H. (2016). Vitamin D and atopic dermatitis: A systematic review and meta-analysis. Nutrition.

