# Supplementary material for: Vitamin D Supplementation and Allergic Diseases during Childhood: A Systematic Review and Meta-Analysis
Source: Nutrients. 2022 Sep 23;14(19):3947. doi: 10.3390/nu14193947 (PMC9571357; doi:10.3390/nu14193947)
Supplement: Supplementary file 1 [file nutrients-14-03947-s001.zip › nutrients-1901218-supplementary.pdf]

Supplementary Online Content

Table S1. Search strategies

Table S2. GRADE assessment (Summary of Findings table)

Table S3. Subgroup analyses of number of children with one or more asthma exacerbations

Table S4. Subgroup analyses of change in forced expiratory volume in the first second (FEV_1) % predicted

Table S5. Subgroup analyses of change in FEV_1/forced vital capacity (FVC) ratio

Table S6. Subgroup analyses of change in total IgE

Table S7. Subgroup analyses of atopic dermatitis severity

Table S8. Adverse events reported in the included RCTs

Figure S1. Assessment on risk of bias for included RCTs

Figure S2. Meta analyses of secondary outcomes among children with asthma

Figure S3. Sensitivity analyses of number of children with one or more asthma exacerbations

Figure S4. Publication bias of number of children with one or more asthma exacerbations

Figure S5. Sensitivity analyses of atopic dermatitis severity

Figure S6. Meta analyses of change in serum 25(OH)D concentration before and after intervention

Figure S7. Meta analyses of post-intervention serum 25(OH)D concentration in vitamin D group

Appendix S1. Methods

Table S1. Search strategies

**PubMed（N=198）**
#1. "Vitamin D"[MeSH]
#2. "Vitamin D"[Title/Abstract]
#3. "hydroxyvitamin D" [Title/Abstract]
#4. "1,25-dihydroxycholecalciferol" [Title/Abstract]
#5. "24,25-dihydroxycholecalciferol" [Title/Abstract]
#6. "1,25-dihydroxyvitamin D3" [Title/Abstract]
#7. "25-hydroxyvitamin D"[Title/Abstract]
#8. "25(OH)D"[Title/Abstract]
#9. "cholecalciferol*"[Title/Abstract]
#10. "calciferol*"[Title/Abstract]
#11. "calcitriol"[Title/Abstract]
#12. "ergocalciferol*"[Title/Abstract]
#13. OR/ #1-#12
#14. "Infant"[Mesh]
#15. "Child"[Mesh]
#16. "Pediatrics"[Mesh]
#17. "Adolescent"[Mesh]
#18. "pediatric*"[Title/Abstract]
#19. "paediatric*"[Title/Abstract]
#20. "child*"[Title/Abstract]
#21. "infant*"[Title/Abstract]
#22. "adolescen*"[Title/Abstract]
#23. "neonat*"[Title/Abstract]
#24. "newborn*"[Title/Abstract]
#25. "teenager*"[Title/Abstract]
#26. "youth*"[Title/Abstract]
#27. "Teen"[Title/Abstract]
#28. "Teens"[Title/Abstract]
#29. "preschool*"[Title/Abstract]
#30. "juvenile"[Title/Abstract]
#31. "baby"[Title/Abstract]
#32. "babies"[Title/Abstract]
#33. "kid"[Title/Abstract]
#34. "kids"[Title/Abstract]
#35. "toddler*"[Title/Abstract]
#36. "schoolage*"[Title/Abstract]
#37. "schoolchild*"[Title/Abstract]
#38. "school age*"[Title/Abstract]
#39. OR/ #14-#38
#40. "Hypersensitivity"[Mesh]
#41. "Asthma"[Mesh]
#42. "Bronchoconstriction"[Mesh]

#43. "Eczema"[Mesh]
#44. "Rhinitis, Allergic"[Mesh]
#45. "Dermatitis, Atopic"[Mesh]
#46. "Anaphylaxis"[Mesh]
#47. "hypersensitiv*"[Title/Abstract]
#48. "allerg*"[Title/Abstract]
#49. "asthma*"[Title/Abstract]
#50. "wheez*"[Title/Abstract]
#51. "eczema*"[Title/Abstract]
#52. "dermatit*"[Title/Abstract]
#53. "rhinit*"[Title/Abstract]
#54. "bronchial spasm"[Title/Abstract]
#55. "bronchospas*"[Title/Abstract]
#56. "bronchoconstrict*"[Title/Abstract]
#57. "bronchial hyperreactivity"[Title/Abstract]
#58. "airway hyperresponsibility"[Title/Abstract]
#59. "atopic sensitization"[Title/Abstract]
#60. "hay fever"[Title/Abstract]
#61. "anaphylaxis"[Title/Abstract]
#62. "anaphylactic reaction*"[Title/Abstract]
#63. "anaphylactic shock"[Title/Abstract]
#64. "anaphylactoid shock"[Title/Abstract]
#65. "anaphylactoid reaction*"[Title/Abstract]
#66. OR/ #40-#65
#67. #13 AND #39 AND #66 Filters: Clinical Study, Clinical Trial, Comparative Study, Humans

EMBASE（N=697）

#1. 'vitamin d'/exp
#2. 'vitamin d':ab,ti
#3. 'hydroxyvitamin d':ab,ti
#4. '1,25-dihydroxycholecalciferol':ab,ti
#5. '24,25-dihydroxycholecalciferol':ab,ti
#6. '1,25-dihydroxyvitamin d3':ab,ti
#7. '25-hydroxyvitamin d':ab,ti
#8. '25(oh)d':ab,ti
#9. 'cholecalciferol*':ab,ti
#10. 'calciferol*':ab,ti
#11. 'calcitriol':ab,ti
#12. 'ergocalciferol*':ab,ti
#13. OR/ #1- #12
#14. 'infant'/exp
#15. 'child'/exp
#16. 'pediatrics'/exp
#17. 'adolescent'/exp

#18. 'pediatric*':ab,ti
#19. 'paediatric*':ab,ti
#20. 'child*':ab,ti
#21. 'infant*':ab,ti
#22. 'adolescen*':ab,ti
#23. 'neonat*':ab,ti
#24. 'newborn*':ab,ti
#25. 'teenager*':ab,ti
#26. 'youth*':ab,ti
#27. 'teen':ab,ti
#28. 'teens':ab,ti
#29. 'preschool*':ab,ti
#30. 'juvenile':ab,ti
#31. 'baby':ab,ti
#32. 'babies':ab,ti
#33. 'kid':ab,ti
#34. 'kids':ab,ti
#35. 'toddler*':ab,ti
#36. 'schoolage*':ab,ti
#37. 'schoolchild*':ab,ti
#38. 'school age*':ab,ti
#39. OR/ #14- #38
#40. 'hypersensitivity'/exp
#41. 'asthma'/exp
#42. 'bronchoconstriction'/exp
#43. 'eczema'/exp
#44. 'allergic rhinitis'/exp
#45. 'atopic dermatitis'/exp
#46. 'anaphylaxis'/exp
#47. 'hypersensitiv*':ab,ti
#48. 'allerg*':ab,ti
#49. 'asthma*':ab,ti
#50. 'wheez*':ab,ti
#51. 'eczema*':ab,ti
#52. 'dermatit*': ab,ti
#53. 'rhinit*':ab,ti
#54. 'bronchial spasm':ab,ti
#55. 'bronchospas*':ab,ti
#56. 'bronchoconstrict*':ab,ti
#57. 'bronchial hyperreactivity':ab,ti
#58. 'airway hyperresponsibility':ab,ti
#59. 'atopic sensitization':ab,ti
#60. 'hay fever':ab,ti
#61. 'anaphylaxis':ab,ti
#62. 'anaphylactic reaction*':ab,ti

#63. 'anaphylactic shock':ab,ti
#64. 'anaphylactoid shock':ab,ti
#65. 'anaphylactoid reaction*':ab,ti
#66. OR/ #40- #65
#67. #13 AND #39 AND #66
#68. #67 AND ('clinical article'/de OR 'clinical study'/de OR 'clinical trial'/de OR 'clinical trial topic'/de OR 'comparative study'/de OR 'controlled clinical trial'/de OR 'controlled study'/de OR 'randomized controlled trial'/de OR 'randomized controlled trial topic'/de)
#69. #68 AND [medline]/lim
#70. #68 NOT #69

Web of science (N=1154)

#1. "Vitamin D" (Topic)
#2. "hydroxyvitamin D" (Topic)
#3. "1,25-dihydroxycholecalciferol" (Topic)
#4. "24,25-dihydroxycholecalciferol" (Topic)
#5. "1,25-dihydroxyvitamin D3" (Topic)
#6. "25-hydroxyvitamin D" (Topic)
#7. "25(OH)D" (Topic)
#8. "cholecalciferol*" (Topic)
#9. "calciferol*" (Topic)
#10. "calcitriol" (Topic)
#11. "ergocalciferol*" (Topic)
#12. OR/ #1- #11
#13. pediatric* (Topic)
#14. paediatric* (Topic)
#15. child* (Topic)
#16. infant* (Topic)
#17. adolescen* (Topic)
#18. newborn* (Topic)
#19. teenager* (Topic)
#20. youth* (Topic)
#21. Teen (Topic)
#22. Teens (Topic)
#23. preschool* (Topic)
#24. juvenile (Topic)
#25. baby (Topic)
#26. babies (Topic)
#27. kid (Topic)
#28. kids (Topic)
#29. toddler* (Topic)
#30. schoolage* (Topic)
#31. schoolchild* (Topic)
#32. "school age*" (Topic)

#33. OR/ #13- #32<br>#34. hypersensitiv* (Topic)<br>#35. allerg* (Topic)<br>#36. asthma* (Topic)<br>#37. wheez* (Topic)<br>#38. eczema* (Topic)<br>#39. dermatit* (Topic)<br>#40. rhinit* (Topic)<br>#41. "bronchial spasm" (Topic)<br>#42. bronchospas* (Topic)<br>#43. bronchoconstrict* (Topic)<br>#44. "bronchial hyperreactivity" (Topic)<br>#45. "airway hyperresponsibility" (Topic)<br>#46. "atopic sensitization" (Topic)<br>#47. "hay fever" (Topic)<br>#48. anaphylaxis (Topic)<br>#49. "anaphylactic reaction*" (Topic)<br>#50. "anaphylactic shock" (Topic)<br>#51. "anaphylactoid shock" (Topic)<br>#52. "anaphylactoid reaction*" (Topic)<br>#53. OR/ #34- #52<br>#54. #12 AND #33 AND #53 AND **Articles (Document Types)**
**The Cochrane library（N=329）**<br>#1. MeSH descriptor: [Vitamin D] explode all trees<br>#2. ("Vitamin D"):ti,ab,kw<br>#3. ("hydroxyvitamin D"):ti,ab,kw<br>#4. ("1,25-dihydroxycholecalciferol"):ti,ab,kw<br>#5. ("24,25-dihydroxycholecalciferol"):ti,ab,kw<br>#6. ("1,25-dihydroxyvitamin D3"):ti,ab,kw<br>#7. ("25-hydroxyvitamin D"):ti,ab,kw<br>#8. ("25(OH)D"):ti,ab,kw<br>#9. ("cholecalciferol*"):ti,ab,kw<br>#10. ("calciferol*"):ti,ab,kw<br>#11. ("calcitriol"):ti,ab,kw<br>#12. ("ergocalciferol*"):ti,ab,kw<br>#13. OR/ #1-#12<br>#14. MeSH descriptor: [Infant] explode all trees<br>#15. MeSH descriptor: [Child] explode all trees<br>#16. MeSH descriptor: [Pediatrics] explode all trees<br>#17. MeSH descriptor: [Adolescent] explode all trees<br>#18. ("pediatric*"):ti,ab,kw<br>#19. ("paediatric*"):ti,ab,kw<br>#20. ("child*"):ti,ab,kw<br>#21. ("infant*"):ti,ab,kw

#22. ("adolescen*"):ti,ab,kw
#23. ("neonat*"):ti,ab,kw
#24. ("newborn*"):ti,ab,kw
#25. ("teenager*"):ti,ab,kw
#26. ("youth*"):ti,ab,kw
#27. ("Teen"):ti,ab,kw
#28. ("Teens"):ti,ab,kw
#29. ("preschool*"):ti,ab,kw
#30. ("juvenile"):ti,ab,kw
#31. ("baby"):ti,ab,kw
#32. ("babies"):ti,ab,kw
#33. ("kid"):ti,ab,kw
#34. ("kids"):ti,ab,kw
#35. ("toddler*"):ti,ab,kw
#36. ("schoolage*"):ti,ab,kw
#37. ("schoolchild*"):ti,ab,kw
#38. ("school age*"):ti,ab,kw
#39. ("adolescent*"):ti,ab,kw
#40. OR/ #14-#39
#41. MeSH descriptor: [Hypersensitivity] explode all trees
#42. MeSH descriptor: [Asthma] explode all trees
#43. MeSH descriptor: [Bronchoconstriction] explode all trees
#44. MeSH descriptor: [Eczema] explode all trees
#45. MeSH descriptor: [Rhinitis, Allergic] explode all trees
#46. MeSH descriptor: [Dermatitis, Atopic] explode all trees
#47. MeSH descriptor: [Anaphylaxis] explode all trees
#48. ("hypersensitiv*"):ti,ab,kw
#49. ("allerg*"):ti,ab,kw
#50. ("asthma*"):ti,ab,kw
#51. ("wheez*"):ti,ab,kw
#52. ("eczema*"):ti,ab,kw
#53. ("dermatit*"):ti,ab,kw
#54. ("rhinit*"):ti,ab,kw
#55. ("bronchial spasm"):ti,ab,kw
#56. ("bronchospas*"):ti,ab,kw
#57. ("bronchoconstrict*"):ti,ab,kw
#58. ("bronchial hyperreactivity"):ti,ab,kw
#59. ("airway hyperresponsibility"):ti,ab,kw
#60. ("atopic sensitization"):ti,ab,kw
#61. ("hay fever"):ti,ab,kw
#62. ("anaphylaxis"):ti,ab,kw
#63. ("anaphylactic reaction*"):ti,ab,kw
#64. ("anaphylactic shock"):ti,ab,kw
#65. ("anaphylactoid shock"):ti,ab,kw
#66. ("anaphylactoid reaction*"):ti,ab,kw

#67. OR/ #41-#66<br>#13 AND #40 AND #67 Filters: Clinical Trial
**CBM（N=176）**<br>#1. "维生素 D"[不加权:扩展]<br>#2. "维生素 D"[常用字段:智能]<br>#3. "25-（OH）D"[常用字段:智能]<br>#4. "胆钙化醇"[常用字段:智能]<br>#5. "骨化醇"[常用字段:智能]<br>#6. "骨化三醇"[常用字段:智能]<br>#7. "麦角钙化醇"[常用字段:智能]<br>#8. "骨化二醇"[常用字段:智能]<br>#9. OR/ #1-#8<br>#10. "儿童"[常用字段:智能]<br>#11. "幼儿"[常用字段:智能]<br>#12. "婴儿"[常用字段:智能]<br>#13. "新生儿"[常用字段:智能]<br>#14. "青少年"[常用字段:智能]<br>#15. "小儿"[常用字段:智能]<br>#16. OR/ #10-#15<br>#17. "超敏反应"[不加权:扩展]<br>#18. "过敏反应"[不加权:扩展]<br>#19. "哮喘"[不加权:扩展]<br>#20. "支气管收缩"[不加权:扩展]<br>#21. "鼻炎，过敏性"[不加权:扩展]<br>#22. "湿疹，汗疱"[不加权:扩展]<br>#23. "皮炎，特应性"[不加权:扩展]<br>#24. "哮喘"[常用字段:智能]<br>#25. "吼喘"[常用字段:智能]<br>#26. "喘鸣"[常用字段:智能]<br>#27. "哮鸣音"[常用字段:智能]<br>#28. "变应性鼻炎"[常用字段:智能]<br>#29. "蛋白过敏"[常用字段:智能]<br>#30. "喘息"[常用字段:智能]<br>#31. "湿疹"[常用字段:智能]<br>#32. "过敏性鼻炎"[常用字段:智能]<br>#33. "特应性皮炎"[常用字段:智能]<br>#34. "超敏反应"[常用字段:智能]<br>#35. "过敏性反应"[常用字段:智能]<br>#36. "变态反应"[常用字段:智能]<br>#37. "过敏反应"[常用字段:智能]<br>#38. "过敏性疾病"[常用字段:智能]<br>#39. "支气管收缩"[常用字段:智能]<br>#40. "支气管痉挛"[常用字段:智能]<br>#41. "支气管高反应性"[常用字段:智能]<br>#42. "气道高反应性"[常用字段:智能]

#43. "过敏性休克"[常用字段:智能]
#44. "食物过敏"[常用字段:智能]
#45. "牛奶过敏"[常用字段:智能]
#46. "枯草热"[常用字段:智能]
#47. "过敏性鼻结膜炎"[常用字段:智能]
#48. "吸入性过敏"[常用字段:智能]
#49. "致敏反应"[常用字段:智能]
#50. OR/ #17-#49
#51. #9 AND #16 AND #50 [限定：临床研究/随机对照试验]

CNKI（N=158）
#1. "维生素 D"[主题]
#2. "25-（OH）D"[主题]
#3. "胆钙化醇"[主题]
#4. "骨化醇"[主题]
#5. "骨化三醇"[主题]
#6. "麦角钙化醇"[主题]
#7. "骨化二醇"[主题]
#8. OR/ #1-#7
#9. "儿童"[主题]
#10. "幼儿"[主题]
#11. "婴儿"[主题]
#12. "新生儿"[主题]
#13. "青少年"[主题]
#14. "小儿"[主题]
#15. OR/ #9-#14
#16. "哮喘"[主题]
#17. "吼喘"[主题]
#18. "喘鸣"[主题]
#19. "哮鸣音"[主题]
#20. "变应性鼻炎"[主题]
#21. "蛋白过敏"[主题]
#22. "喘息"[主题]
#23. "湿疹"[主题]
#24. "过敏性鼻炎"[主题]
#25. "特应性皮炎"[主题]
#26. "超敏反应"[主题]
#27. "过敏性反应"[主题]
#28. "变态反应"[主题]
#29. "过敏反应"[主题]
#30. "过敏性疾病"[主题]
#31. "支气管收缩"[主题]
#32. "支气管痉挛"[主题]
#33. "支气管高反应性"[主题]
#34. "气道高反应性"[主题]

#35. "过敏性休克"[主题]<br>#36. "食物过敏"[主题]<br>#37. "牛奶过敏"[主题]<br>#38. "枯草热"[主题]<br>#39. "过敏性鼻结膜炎"[主题]<br>#40. "吸入性过敏"[主题]<br>#41. "致敏反应"[主题]<br>#42. OR/ #16-#41<br>#8 AND #15 AND #42 [限定：临床研究]
**Wanfang（N=620）**<br>#1. "维生素 D"[主题]<br>#2. "25-（OH）D"[主题]<br>#3. "胆钙化醇"[主题]<br>#4. "骨化醇"[主题]<br>#5. "骨化三醇"[主题]<br>#6. "麦角钙化醇"[主题]<br>#7. "骨化二醇"[主题]<br>#8. OR/ #1-#7<br>#9. "儿童"[主题]<br>#10. "幼儿"[主题]<br>#11. "婴儿"[主题]<br>#12. "新生儿"[主题]<br>#13. "青少年"[主题]<br>#14. "小儿"[主题]<br>#15. OR/ #9-#14<br>#16. "哮喘"[主题]<br>#17. "吼喘"[主题]<br>#18. "喘鸣"[主题]<br>#19. "哮鸣音"[主题]<br>#20. "变应性鼻炎"[主题]<br>#21. "蛋白过敏"[主题]<br>#22. "喘息"[主题]<br>#23. "湿疹"[主题]<br>#24. "过敏性鼻炎"[主题]<br>#25. "特应性皮炎"[主题]<br>#26. "超敏反应"[主题]<br>#27. "过敏性反应"[主题]<br>#28. "变态反应"[主题]<br>#29. "过敏反应"[主题]<br>#30. "过敏性疾病"[主题]<br>#31. "支气管收缩"[主题]<br>#32. "支气管痉挛"[主题]<br>#33. "支气管高反应性"[主题]<br>#34. "气道高反应性"[主题]<br>#35. "过敏性休克"[主题]

#36. "食物过敏"[主题]
#37. "牛奶过敏"[主题]
#38. "枯草热"[主题]
#39. "过敏性鼻结膜炎"[主题]
#40. "吸入性过敏"[主题]
#41. "致敏反应"[主题]
#42. OR/ #16-#41
#43. #8 AND #15 AND #42

Table S2. GRADE Assessment (Summary of Findings table)

Certainty assessment						№ of patients		Effect		Certainty
№ of studies	Risk of bias	Inconsistency	Indirectness	Imprecision	Other considerations	Vitamin D group	Placebo group	Relative (95% CI)	Absolute (95% CI)	
**Number of children with one or more asthma exacerbations**										
11	not serious	not serious	not serious	serious[a]	none	224/577 (38.8%)	250/566 (44.2%)	RR 0.84 (0.65 to 1.08)	71 fewer per 1,000 (from 155 fewer to 35 more)	⨁⨁⨁◯ Moderate
**Number of children with one or more asthma exacerbations, subgroup with baseline 25(OH)D < 10 ng/mL**										
1	serious[b]	not serious	not serious	serious[c]	none	11/33 (33.3%)	16/23 (69.6%)	RR 0.48 (0.28 to 0.83)	362 fewer per 1,000 (from 501 fewer to 118 fewer)	⨁⨁◯◯ Low
**Number of children requiring systemic corticosteroids for asthma exacerbations**										
5	not serious	not serious	not serious	serious[a]	none	29/133 (21.8%)	32/120 (26.7%)	RR 0.99 (0.68 to 1.46)	3 fewer per 1,000 (from 85 fewer to 123 more)	⨁⨁⨁◯ Moderate
**Number of children requiring emergency department visit or hospital admission, or both for asthma exacerbation**										
5	not serious	not serious	not serious	serious[a]	none	116/232 (50.0%)	112/213 (52.6%)	RR 1.01 (0.91 to 1.12)	5 more per 1,000 (from 47 fewer to 63 more)	⨁⨁⨁◯ Moderate
**Atopic dermatitis severity assessed by EASI or SCORAD**										
8	not serious	serious[d]	not serious	not serious	none	242	241	-	SMD 4.94 lower (6.61 lower to 3.27 lower)	⨁⨁⨁◯ Moderate
**Atopic dermatitis severity assessed by EASI or SCORAD, subgroup with baseline 25(OH)D < 30 ng/mL**										

4	not serious	not serious	not serious	serious[c]	none	105	106	-	MD 4.03 lower (6.39 lower to 1.68 lower)	⊕⊕⊕◯ Moderate
**Number of children with serious adverse events**										
6	not serious	not serious	not serious	serious[a]	none	1/201 (0.5%)	1/175 (0.6%)	RR 0.82 (0.09 to 7.68)	1 fewer per 1,000 (from 5 fewer to 38 more)	⊕⊕⊕◯ Moderate

a. 95% confidence interval crosses the null line

b. High risk of bias in blinding of participants and researchers

c. Small sample size

d. Differences in direction and large heterogeneity

-. Not applicable

Table S3. Subgroup analyses of number of children with one or more asthma exacerbations

Variable	No. of trials	No. of participants	Vitamin D group (event/total)	Placebo group (event/total)	Risk Ratio (95% CI)	I^2, %	$P_{interaction}$
**Baseline vitamin D level**							
≥ 30 ng/ml	0	NA	NA	NA	NA	NA	0.04*
20-29 ng/ml	2	253	51/125	48/128	1.10 (0.81, 1.48)	0	
10-19 ng/ml	3	379	71/189	75/190	0.96 (0.74, 1.24)	9	
< 10 ng/ml	1	56	11/33	16/23	0.48 (0.28, 0.83)	NA	
**Age**							
1-5 years	2	69	20/34	19/35	1.05 (0.59, 1.85)	38	0.15
≥ 5 years	7	634	66/320	101/314	0.60 (0.37, 0.97)	55	
**Treatment duration**							
< 6 months	4	294	12/150	27/144	0.56 (0.28, 1.13)	13	0.22
≥ 6 months	7	849	212/427	223/422	0.90 (0.70, 1.16)	63	
**Daily dose of vitamin D**							
< 2000 IU	7	756	165/386	172/370	0.92 (0.67, 1.25)	52	0.43
≥ 2000 IU	4	387	59/191	78/196	0.72 (0.44, 1.20)	60	
**Bolus-dose vitamin D given**							
No	7	743	90/377	102/366	0.79 (0.52, 1.20)	47	0.85
Yes	4	400	134/200	148/200	0.84 (0.54, 1.30)	77	
**Cotreatment with inhaled corticosteroids**							
No	7	800	165/406	187/394	0.77 (0.53, 1.13)	68	0.64
Yes	4	343	59/171	63/172	0.89 (0.55, 1.44)	54	

Note: *: indicated that baseline serum 25[OH]D levels modified the effects of vitamin D supplementation on asthma exacerbation risk. A significant overlap in severity of asthma precluded the subgroup analysis on this variable. NA: not applicable

Table S4. Subgroup analyses of change in forced expiratory volume in the first second (FEV_1) % predicted

Variable	No. of trials	No. of participants	Mean difference (95% CI)	I^2, %	$P_{interaction}$
**Baseline vitamin D level**					
≥ 30 ng/ml	0	NA	NA	NA	NA
20-29 ng/ml	0	NA	NA	NA	
10-19 ng/ml	2	215	-0.77 (-8.96, 6.75)	46	
< 10 ng/ml	0	NA	NA	NA	
**Age**					
1-5 years	0	NA	NA	NA	NA
≥ 5 years	7	397	0.03 (-3.22, 3.27)	55	
**Treatment duration**					
< 6 months	3	128	-2.93 (-6.65, 0.78)	48	0.03
≥ 6 months	4	269	3.12 (-0.67, 6.90)	15	
**Daily dose of vitamin D**					
< 2000 IU	5	303	1.63 (-1.52, 4.78)	37	0.006
≥ 2000 IU	2	94	-5.36 (-9.30, -1.43)*	0	
**Bolus-dose vitamin D given**					
No	7	397	0.03 (-3.22, 3.27)	55	NA
Yes	0	NA	NA	NA	
**Cotreatment with inhaled corticosteroids**					
No	4	259	2.05 (-4.33, 8.44)	76	0.40
Yes	3	138	-0.85 (-3.19, 1.48)	0	

Note: *: Although there was a significant improvement in FEV_1% in the placebo group as compared to the ≥ 2000 IU daily dose of vitamin D group, we cannot conclude that vitamin D supplementation was related to reduced FEV_1% because the sample size of the original studies was too small and the baseline FEV_1% in both groups were high, leaving little potential for improvement. A significant overlap in severity of asthma precluded the subgroup analysis on this variable. NA: not applicable

Table S5. Subgroup analyses of change in FEV_1/forced vital capacity (FVC) ratio

Variable	No. of trials	No. of participants	Mean difference (95% CI)	I^2, %	$P_{interaction}$
**Baseline vitamin D level**					
≥ 30 ng/ml	0	NA	NA	NA	NA
20-29 ng/ml	0	NA	NA	NA	
10-19 ng/ml	2	225	1.33 (-0.71, 3.36)	0	
< 10 ng/ml	0	NA	NA	NA	
**Age**					
1-5 years	0	NA	NA	NA	NA
≥ 5 years	3	263	-0.02 (-3.03, 3.00)	68	
**Treatment duration**					
< 6 months	2	104	-0.40 (-5.69, 4.89)	81	0.69
≥ 6 months	1	159	0.80 (-1.68, 3.28)	NA	
**Daily dose of vitamin D**					
< 2000 IU	1	159	0.80 (-1.68, 3.28)	NA	0.69
≥ 2000 IU	2	104	-0.40 (-5.69, 4.89)	81	
**Bolus-dose vitamin D given**					
No	3	263	-0.02 (-3.03, 3.00)	68	NA
Yes	0	NA	NA	NA	
**Cotreatment with inhaled corticosteroids**					
No	3	263	-0.02 (-3.03, 3.00)	68	NA
Yes	0	NA	NA	NA	

Note: A significant overlap in severity of asthma precluded the subgroup analysis on this variable. NA: not applicable

Table S6. Subgroup analyses of change in total IgE

Variable	No. of trials	No. of participants	Mean difference (95% CI)	I^2, %	$P_{interaction}$
**Baseline vitamin D level**					
≥ 30 ng/ml	0	NA	NA	NA	NA
20-29 ng/ml	1	174	0.00 (-0.30, 0.30)	NA	
10-19 ng/ml	0	NA	NA	NA	
< 10 ng/ml	0	NA	NA	NA	
**Age**					
1-5 years	0	NA	NA	NA	NA
≥ 5 years	3	244	-0.06 (-0.31, 0.19)	0	
**Treatment duration**					
< 6 months	1	38	-0.27 (-0.91, 0.37)	NA	0.49
≥ 6 months	2	206	-0.02 (-0.30, 0.25)	0	
**Daily dose of vitamin D**					
< 2000 IU	1	32	-0.15 (-0.84, 0.55)	NA	0.79
≥ 2000 IU	2	212	-0.05 (-0.32, 0.22)	0	
**Bolus-dose vitamin D given**					
No	3	244	-0.06 (-0.31, 0.19)	0	NA
Yes	0	NA	NA	NA	
**Cotreatment with inhaled corticosteroids**					
No	2	70	-0.21 (-0.68, 0.26)	0	0.45
Yes	1	174	0.00 (-0.30, 0.30)	NA	

Note: A significant overlap in severity of asthma precluded the subgroup analysis on this variable. NA: not applicable

Table S7. Subgroup analyses of atopic dermatitis severity

Variable	No. of trials	No. of participants	Std. Mean Difference (95% CI)	I^2, %	$P_{interaction}$
**Baseline vitamin D level**					
< 30 ng/ml	4	211	-0.40 (-0.67, -0.13)	0	0.07
≥ 30 ng/ml	1	89	0.07 (-0.35, 0.48)	NA	
**Daily dose of vitamin D**					
<2000 IU	5	314	-0.60 (-1.10, -0.10)	76	0.41
≥2000 IU	3	169	-0.29 (-0.82, 0.23)	59	
**Bolus-dose vitamin D given**					
No	7	423	-0.54 (-0.97, -0.10)	76	0.47
Yes	1	60	-0.29 (-0.79, 0.22)	NA	

Note: The lowest limit of baseline serum 25(OH)D was less than 10 ng/mL in the studies that only recruited children with baseline serum 25(OH)D of 30 ng/mL. Therefore, we were unable to investigate effects in subgroups defined by the 10 ng/mL and 20 ng/mL cutoffs for baseline serum 25(OH)D level. As a result, we only examined subgroups with baseline 25(OH)D levels of < 30ng/mL and ≥ 30ng/mL. We were unable to separate the study population into two groups using the cut-off value of 5 years of age for subgroup analysis since the majority of studies included both children under and over 5 years of age. The longest trials were only of three months duration hence the effect of longer-term supplementation could not be analyzed. A significant overlap in severity of asthma and cotreatment precluded the subgroup analyses on these variables.

Table S8. Adverse events reported in the included RCTs

Adverse events	No. of trials	No. of participants	Vitamin D group (event/total)	Placebo group (event/total)	Risk ratio (95% CI)	I^2
Rash	3	310	14/156	11/154	1.10 (0.57, 2.15)	2
Gastrointestinal disorders						
Constipation	1	250	12/125	11/125	1.09 (0.50, 2.38)	NA
Nausea	1	250	8/125	7/125	1.14 (0.43, 3.06)	NA
Pain abdomen	1	250	40/125	41/125	0.98 (0.68, 1.40)	NA
Vomiting	1	250	34/125	28/125	1.21 (0.79, 1.87)	NA
Nervous system disorders						
Headache	1	250	25/125	25/125	1.00 (0.61, 1.64)	NA
Seizures	1	250	0/125	1/125	0.33 (0.01, 8.10)	NA
Blood and lymphatic system disorders	1	47	3/23	1/24	3.13 (0.35, 27.96)	NA
Ear and labyrinth disorders	1	47	0/23	2/24	0.21 (0.01, 4.12)	NA
Eye disorders	1	47	0/23	1/24	0.35 (0.01, 8.11)	NA
General disorders	1	47	13/23	17/24	0.80 (0.51, 1.24)	NA
Immune system disorders	1	47	2/23	5/24	0.42 (0.09, 1.94)	NA
Musculoskeletal disorders	1	47	0/23	1/24	0.35 (0.01, 8.11)	NA
Infections	1	47	19/23	17/24	1.17 (0.85, 1.60)	NA
Respiratory, thoracic and mediastinal disorders	1	47	4/23	7/24	0.60 (0.20, 1.77)	NA
Reproductive system and breast disorders	1	47	1/23	0/24	3.13 (0.13, 73.01)	NA
Serious adverse events	6	376	1/201	1/175	0.82 (0.09, 7.68)	0

NA: not applicable. Only one study was included, therefore I^2 was not applicable.

Figure S1. Assessment on risk of bias for included RCTs

A. Risk of bias summary

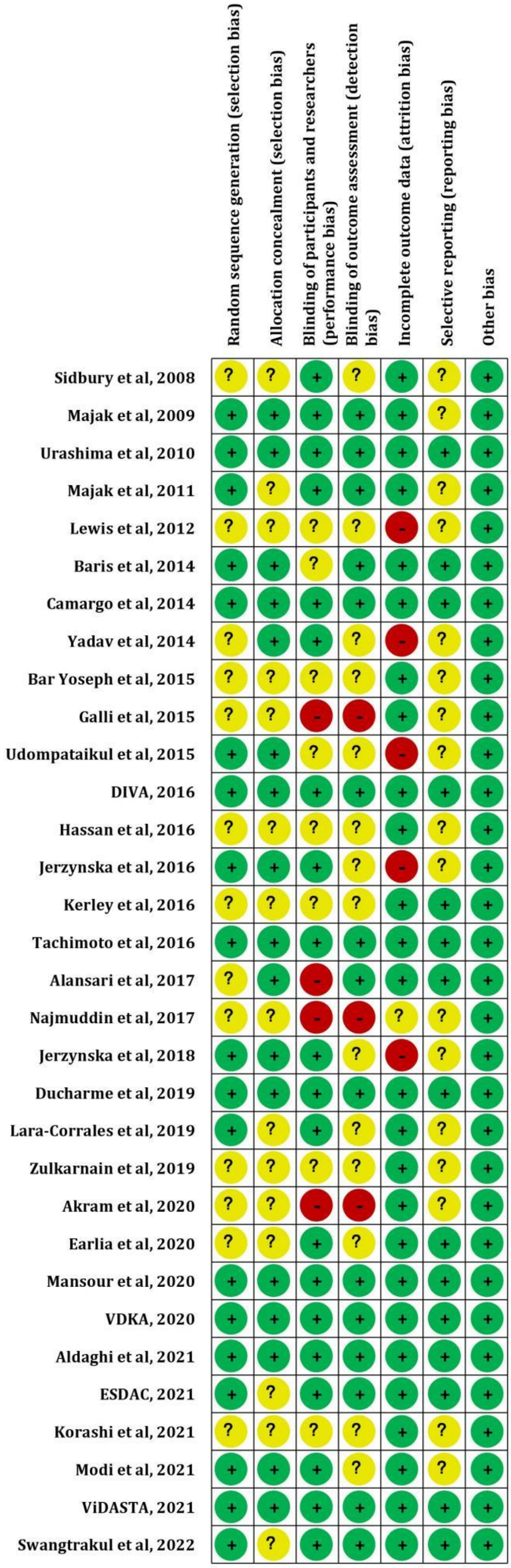

B. Risk of bias graph

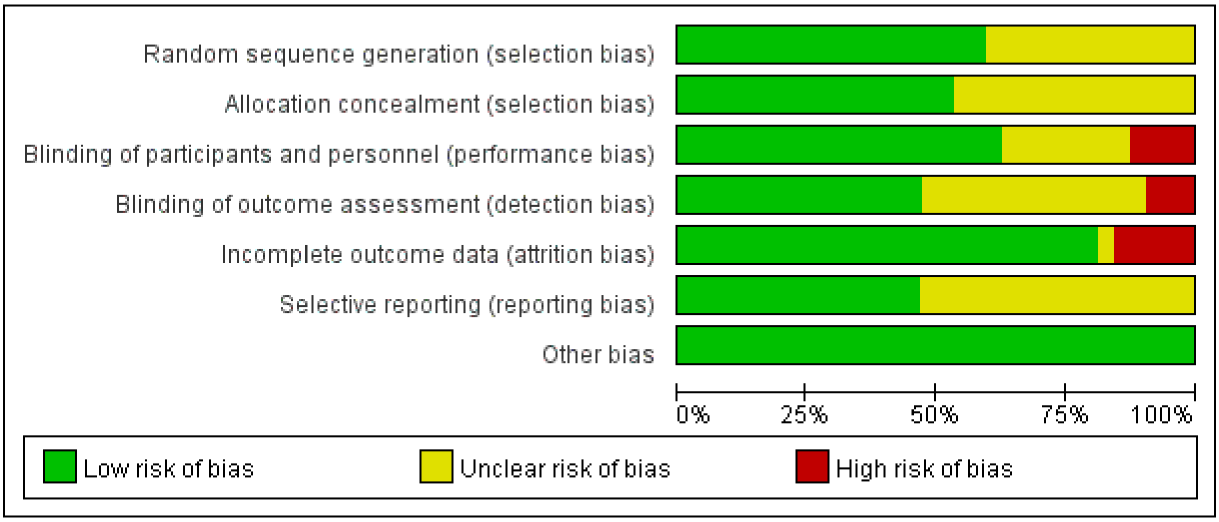

Figure S2. Meta analyses of secondary outcomes among children with asthma

A. Number of children requiring systemic corticosteroids for asthma exacerbations

Study or Subgroup	Vitamin D Events	Vitamin D Total	Placebo Events	Placebo Total	Weight	Risk Ratio M-H, Random, 95% CI
DIVA, 2016	5	11	7	11	24.0%	0.71 [0.33, 1.57]
Ducharme et al, 2019	14	23	10	24	44.9%	1.46 [0.82, 2.60]
Kerley et al, 2016	5	17	8	22	17.5%	0.81 [0.32, 2.03]
Tachimoto et al, 2016	1	54	0	35	1.5%	1.96 [0.08, 46.89]
ViDASTA, 2021	4	28	7	28	12.0%	0.57 [0.19, 1.74]
**Total (95% CI)**		**133**		**120**	**100.0%**	**0.99 [0.68, 1.46]**
Total events	29		32			

Heterogeneity: Tau² = 0.00; Chi² = 3.75, df = 4 (P = 0.44); I² = 0%
Test for overall effect: Z = 0.03 (P = 0.98)

Risk Ratio M-H, Random, 95% CI

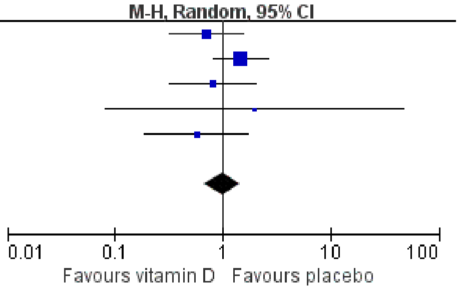

B. Number of children requiring emergency department visit or hospitalization, or both

Study or Subgroup	Vitamin D Events	Vitamin D Total	Placebo Events	Placebo Total	Weight	Risk Ratio M-H, Random, 95% CI
Alansari et al, 2017	100	116	99	115	95.7%	1.00 [0.90, 1.11]
DIVA, 2016	0	11	1	11	0.1%	0.33 [0.02, 7.39]
Ducharme et al, 2019	15	23	12	24	4.1%	1.30 [0.79, 2.15]
Tachimoto et al, 2016	1	54	0	35	0.1%	1.96 [0.08, 46.89]
ViDASTA, 2021	0	28	0	28		Not estimable
**Total (95% CI)**		**232**		**213**	**100.0%**	**1.01 [0.91, 1.12]**
Total events	116		112			

Heterogeneity: Tau² = 0.00; Chi² = 1.80, df = 3 (P = 0.61); I² = 0%
Test for overall effect: Z = 0.23 (P = 0.82)

Risk Ratio M-H, Random, 95% CI

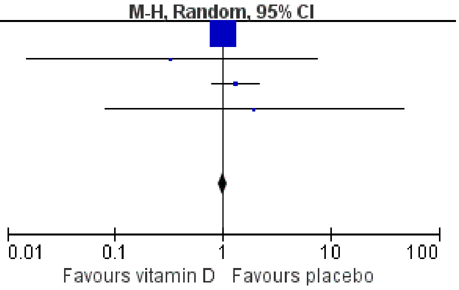

C. Change in childhood asthma control test (C-ACT) score

Study or Subgroup	Vitamin D Mean	Vitamin D SD	Vitamin D Total	Placebo Mean	Placebo SD	Placebo Total	Weight	Mean Difference IV, Random, 95% CI
ESDAC, 2021	2.4	3.8	112	1.9	3	108	29.0%	0.50 [-0.40, 1.40]
Kerley et al, 2016	1.3	4.9	17	2.8	4	22	15.6%	-1.50 [-4.37, 1.37]
Tachimoto et al, 2016	1.2	1.9	54	0	1.5	34	30.1%	1.20 [0.49, 1.91]
ViDASTA, 2021	6.5	2.7	28	8.9	2.8	28	25.3%	-2.40 [-3.84, -0.96]
**Total (95% CI)**			**211**			**192**	**100.0%**	**-0.34 [-1.92, 1.25]**

Heterogeneity: Tau² = 2.03; Chi² = 21.04, df = 3 (P = 0.0001); I² = 86%
Test for overall effect: Z = 0.42 (P = 0.68)

Mean Difference IV, Random, 95% CI

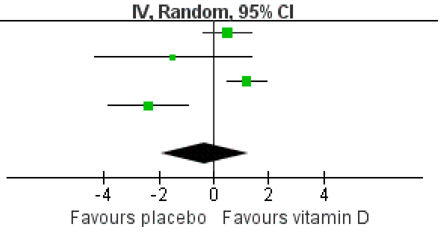

D. Change in total asthma symptom (TAS) score

Study or Subgroup	Vitamin D Mean	Vitamin D SD	Vitamin D Total	Placebo Mean	Placebo SD	Placebo Total	Weight	Mean Difference IV, Random, 95% CI
Baris et al, 2014	-0.6	0.53	17	-1.2	1.05	15	37.5%	0.60 [0.01, 1.19]
Majak et al, 2009	-0.1	0.05	17	-0.1	0.04	17	62.5%	0.00 [-0.03, 0.03]
**Total (95% CI)**			**34**			**32**	**100.0%**	**0.23 [-0.34, 0.79]**

Heterogeneity: Tau² = 0.13; Chi² = 3.99, df = 1 (P = 0.05); I² = 75%
Test for overall effect: Z = 0.78 (P = 0.44)

Mean Difference IV, Random, 95% CI

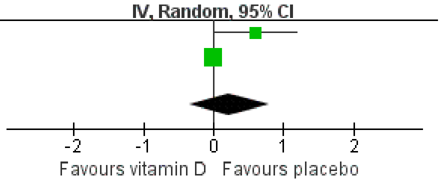

E. Change in forced expiratory volume in the first second (FEV_1) % predicted

Study or Subgroup	Vitamin D Mean	Vitamin D SD	Vitamin D Total	Placebo Mean	Placebo SD	Placebo Total	Weight	Mean Difference IV, Random, 95% CI
Baris et al, 2014	6	14.5	17	1	12.3	15	8.7%	5.00 [-4.29, 14.29]
ESDAC, 2021	4.7	15.5	84	2.9	14.4	75	18.5%	1.80 [-2.85, 6.45]
Kerley et al, 2016	-3.7	4.3	17	1.5	8.6	21	19.9%	-5.20 [-9.41, -0.99]
Lewis et al, 2012	13.5	14.2	15	2.7	12.9	15	8.1%	10.80 [1.09, 20.51]
Majak et al, 2009	0.7	4.5	17	1.4	2.9	17	25.3%	-0.70 [-3.24, 1.84]
Majak et al, 2011	4.6	12.2	24	4.4	12.1	24	12.8%	0.20 [-6.67, 7.07]
ViDASTA, 2021	21.2	23.2	28	27.7	18.8	28	6.7%	-6.50 [-17.56, 4.56]
**Total (95% CI)**			**202**			**195**	**100.0%**	**0.03 [-3.22, 3.27]**

Heterogeneity: Tau² = 9.17; Chi² = 13.46, df = 6 (P = 0.04); I² = 55%
Test for overall effect: Z = 0.02 (P = 0.99)

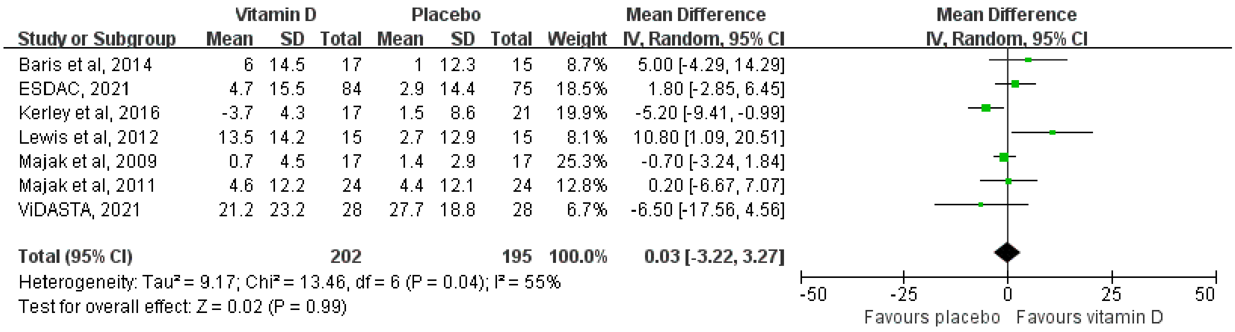

F. Change in FEV_1/forced vital capacity (FVC) ratio

Study or Subgroup	Vitamin D Mean	Vitamin D SD	Vitamin D Total	Placebo Mean	Placebo SD	Placebo Total	Weight	Mean Difference IV, Random, 95% CI
ESDAC, 2021	-1.7	9.1	84	-2.5	6.8	75	36.9%	0.80 [-1.68, 3.28]
Kerley et al, 2016	-2	5.1	17	1	3.7	21	33.8%	-3.00 [-5.90, -0.10]
Najmuddin et al, 2017	3	8.4	33	0.6	6.1	33	29.3%	2.40 [-1.14, 5.94]
**Total (95% CI)**			**134**			**129**	**100.0%**	**-0.02 [-3.03, 3.00]**

Heterogeneity: Tau² = 4.81; Chi² = 6.27, df = 2 (P = 0.04); I² = 68%
Test for overall effect: Z = 0.01 (P = 0.99)

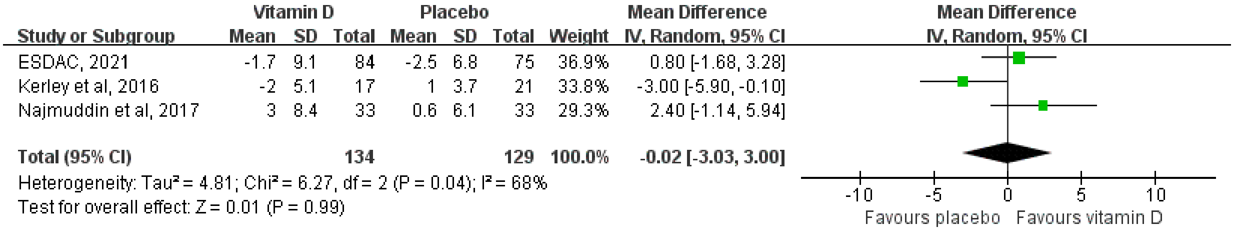

G. Change in forced expiratory flow between 25% and 75% of vital capacity (FEF_{25-75})

Study or Subgroup	Vitamin D Mean	Vitamin D SD	Vitamin D Total	Placebo Mean	Placebo SD	Placebo Total	Weight	Mean Difference IV, Random, 95% CI
Baris et al, 2014	3	21	17	9	22.9	15	27.1%	-6.00 [-21.30, 9.30]
ESDAC, 2021	3.5	30.5	84	2.8	29.5	75	72.9%	0.70 [-8.63, 10.03]
**Total (95% CI)**			**101**			**90**	**100.0%**	**-1.12 [-9.09, 6.85]**

Heterogeneity: Tau² = 0.00; Chi² = 0.54, df = 1 (P = 0.46); I² = 0%
Test for overall effect: Z = 0.28 (P = 0.78)

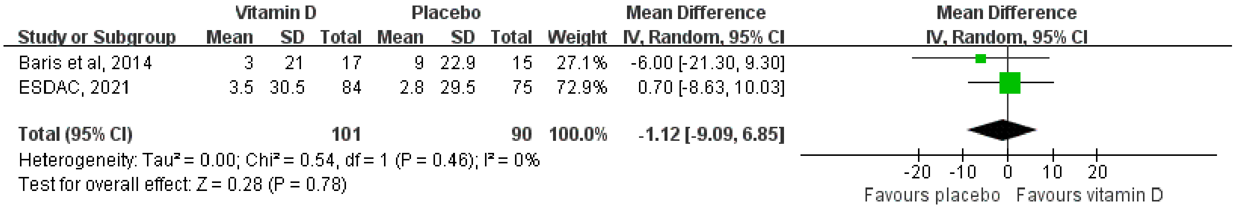

H. Change in fractional exhaled nitric oxide (FeNO)

Study or Subgroup	Vitamin D Mean	Vitamin D SD	Vitamin D Total	Placebo Mean	Placebo SD	Placebo Total	Weight	Mean Difference IV, Random, 95% CI
Bar Yoseph et al, 2015	-2.4	34.6	19	-7.6	49.1	19	11.2%	5.20 [-21.81, 32.21]
ViDASTA, 2021	-1.9	13.3	28	-10	22.2	28	88.8%	8.10 [-1.49, 17.69]
**Total (95% CI)**			**47**			**47**	**100.0%**	**7.78 [-1.26, 16.81]**

Heterogeneity: Tau² = 0.00; Chi² = 0.04, df = 1 (P = 0.84); I² = 0%
Test for overall effect: Z = 1.69 (P = 0.09)

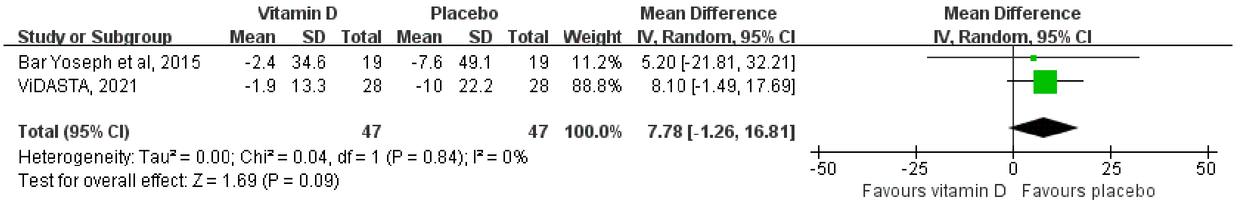

I. Change in total IgE

Study or Subgroup	Vitamin D Mean	SD	Total	Placebo Mean	SD	Total	Weight	Std. Mean Difference IV, Random, 95% CI
Baris et al, 2014	89	615.8	17	187	667.4	15	13.1%	-0.15 [-0.84, 0.55]
Bar Yoseph et al, 2015	-34.7	441.6	19	105.7	579.1	19	15.5%	-0.27 [-0.91, 0.37]
VDKA, 2020	0	4	84	0	6.3	90	71.5%	0.00 [-0.30, 0.30]
**Total (95% CI)**			**120**			**124**	**100.0%**	**-0.06 [-0.31, 0.19]**

Heterogeneity: Tau² = 0.00; Chi² = 0.62, df = 2 (P = 0.73); I² = 0%
Test for overall effect: Z = 0.47 (P = 0.64)

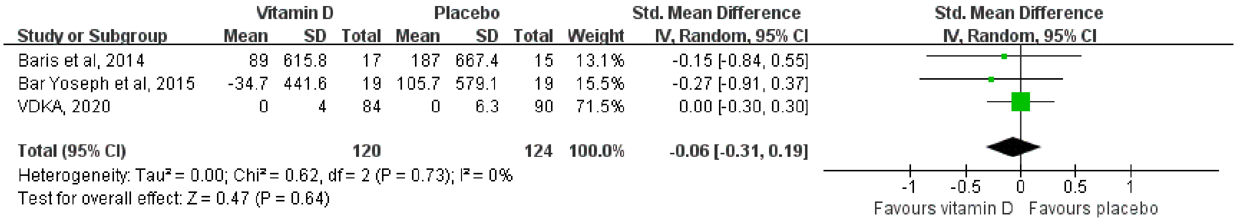

J. Change in interleukin 10 (IL-10)

Study or Subgroup	Vitamin D Mean	SD	Total	Placebo Mean	SD	Total	Weight	Mean Difference IV, Random, 95% CI
Baris et al, 2014	26	15.1	17	31.6	9.1	15	26.7%	-5.60 [-14.13, 2.93]
El-Korashi et al, 2021	29	30.5	23	12.8	29.8	23	23.3%	16.20 [-1.23, 33.63]
Kerley et al, 2016	-11.6	22.6	17	-16.6	15.1	22	25.4%	5.00 [-7.46, 17.46]
Majak et al, 2009	-61.4	17.8	16	-20.1	24.9	17	24.5%	-41.30 [-56.00, -26.60]
**Total (95% CI)**			**73**			**77**	**100.0%**	**-6.57 [-27.15, 14.01]**

Heterogeneity: Tau² = 393.41; Chi² = 31.35, df = 3 (P < 0.00001); I² = 90%
Test for overall effect: Z = 0.63 (P = 0.53)

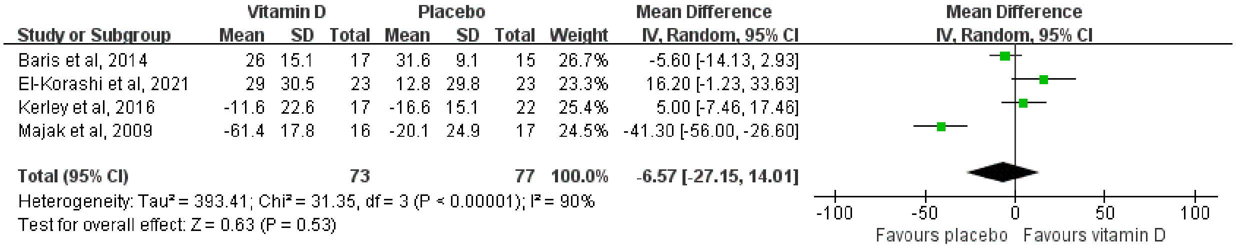

Figure S3. Sensitivity analyses of number of children with one or more asthma exacerbations

A. Excluding trials assessed as being at high risk of bias in any domains

Study or Subgroup	Vitamin D Events	Vitamin D Total	Placebo Events	Placebo Total	Weight	Risk Ratio M-H, Random, 95% CI
DIVA, 2016	5	11	7	11	10.7%	0.71 [0.33, 1.57]
Ducharme et al, 2019	15	23	12	24	17.9%	1.30 [0.79, 2.15]
ESDAC, 2021	38	107	31	102	21.7%	1.17 [0.79, 1.72]
Kerley et al, 2016	5	17	8	22	8.5%	0.81 [0.32, 2.03]
Majak et al, 2011	4	24	11	24	7.6%	0.36 [0.13, 0.98]
Tachimoto et al, 2016	1	54	0	35	0.9%	1.96 [0.08, 46.89]
Urashima et al, 2010	2	51	12	59	4.1%	0.19 [0.05, 0.82]
VDKA, 2020	36	96	33	96	22.1%	1.09 [0.75, 1.59]
ViDASTA, 2021	4	28	7	28	6.4%	0.57 [0.19, 1.74]
**Total (95% CI)**		**411**		**401**	**100.0%**	**0.88 [0.64, 1.20]**
Total events	110		121			

Heterogeneity: Tau² = 0.08; Chi² = 13.34, df = 8 (P = 0.10); I² = 40%
Test for overall effect: Z = 0.80 (P = 0.43)

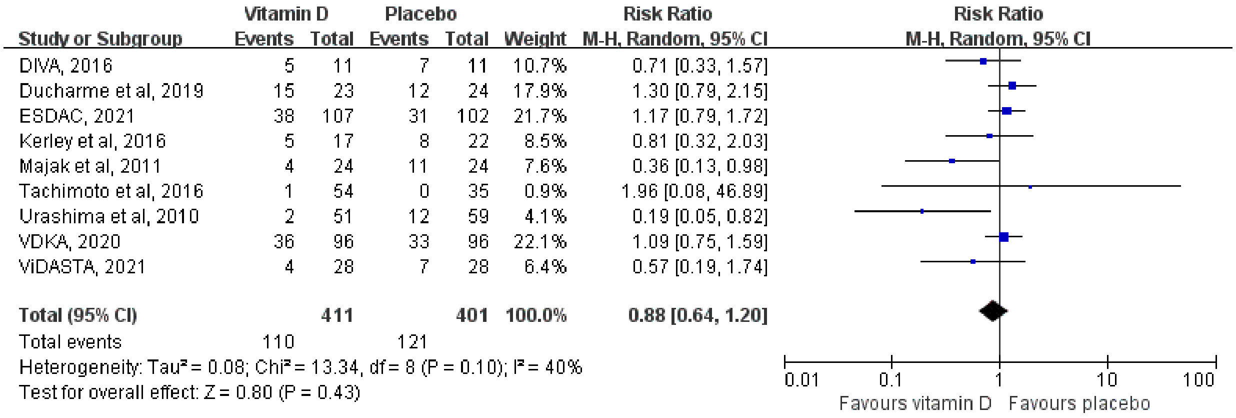

B. Excluding trials in which mean or SD, or both of them were imputed for missing data

We were unable to perform this sensitivity analysis because the effect estimates of the incidence of asthma exacerbation were not reported as mean (SD), which eliminated the requirement to impute missing data.

Figure S4. Publication bias of number of children with one or more asthma exacerbations

A. Funnel plot of number of children with one or more asthma exacerbations

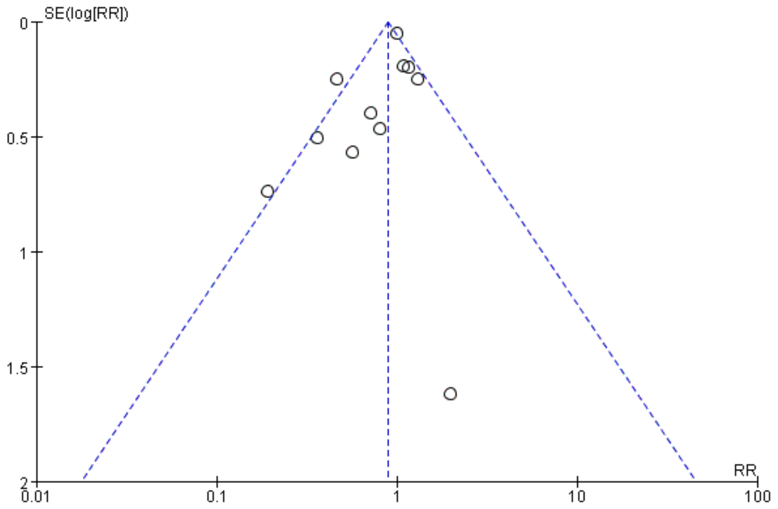

B. Egger's test of of number of children with one or more asthma exacerbations

Number of studies = 11 Root MSE = 1.358

Std_Eff	Coef.	Std. Err.	t	P>|t|	[95% Conf. Interval]	
slope	.064967	.0845103	0.77	0.462	-.1262086	.2561426
bias	-.8950571	.5477642	-1.63	**0.137**	-2.134186	.3440716

Test of H0: no small-study effects **P = 0.137**

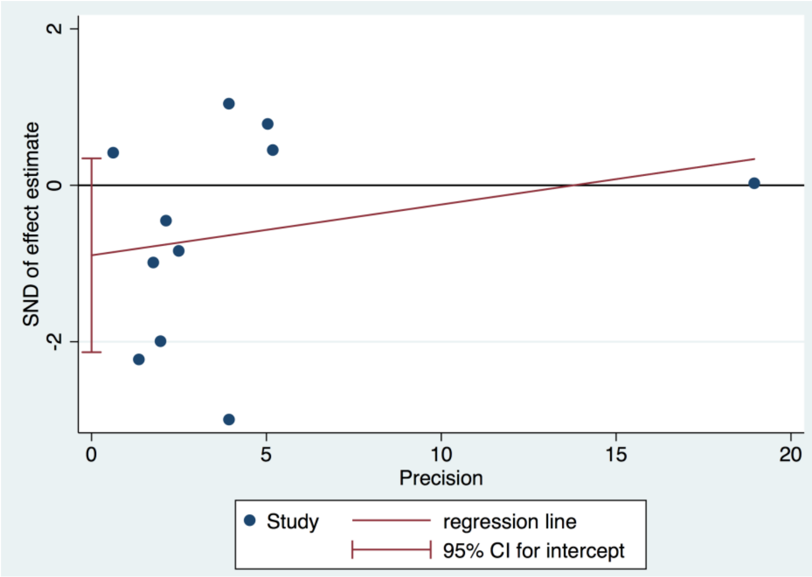

Figure S5. Sensitivity analyses of atopic dermatitis severity

A. Excluding trials assessed as being at high risk of bias in any domains

	Vitamin D			Placebo				Std. Mean Difference
Study or Subgroup	Mean	SD	Total	Mean	SD	Total	Weight	IV, Random, 95% CI
Camargo et al, 2014	-6.5	8.8	57	-3.3	7.6	47	20.3%	-0.38 [-0.77, 0.01]
Corrales et al, 2019	-11.9	15.4	21	-9.3	13.4	24	16.6%	-0.18 [-0.76, 0.41]
Earlia et al, 2020	-19.3	10.7	34	-3.6	8.6	34	17.3%	-1.60 [-2.15, -1.05]
Mansour et al, 2020	-24	12.7	44	-18.9	8.8	42	19.6%	-0.46 [-0.89, -0.03]
Modi et al, 2021	-44.2	6.7	30	-41.9	9	30	18.1%	-0.29 [-0.79, 0.22]
Sidbury et al, 2008	-4.6	8.9	5	-2.2	8.9	6	8.1%	-0.25 [-1.44, 0.95]
**Total (95% CI)**			**191**			**183**	**100.0%**	**-0.55 [-0.96, -0.13]**

Heterogeneity: Tau² = 0.18; Chi² = 17.63, df = 5 (P = 0.003); I² = 72%
Test for overall effect: Z = 2.57 (P = 0.01)

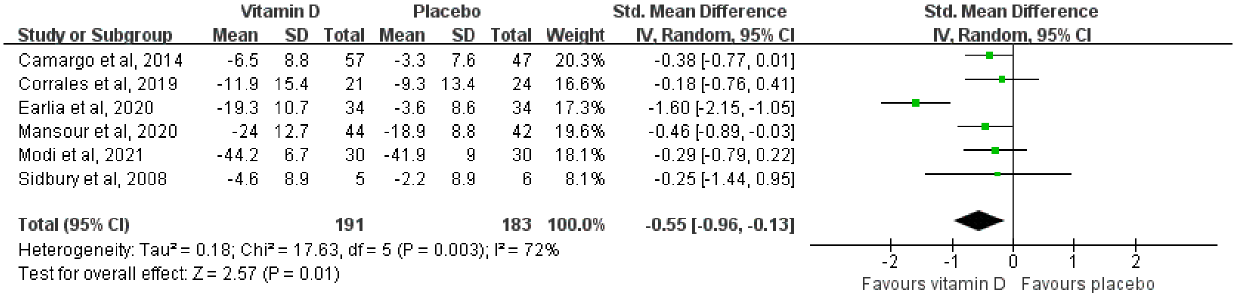

B. Excluding trials in which mean or SD, or both of them were imputed for missing data

	Vitamin D			Placebo				Std. Mean Difference
Study or Subgroup	Mean	SD	Total	Mean	SD	Total	Weight	IV, Random, 95% CI
Camargo et al, 2014	-6.5	8.8	57	-3.3	7.6	47	18.0%	-0.38 [-0.77, 0.01]
Corrales et al, 2019	-11.9	15.4	21	-9.3	13.4	24	15.1%	-0.18 [-0.76, 0.41]
Earlia et al, 2020	-19.3	10.7	34	-3.6	8.6	34	15.6%	-1.60 [-2.15, -1.05]
Galli et al, 2015	-0.2	11.1	41	-1.3	19.4	48	17.6%	0.07 [-0.35, 0.48]
Mansour et al, 2020	-24	12.7	44	-18.9	8.8	42	17.4%	-0.46 [-0.89, -0.03]
Modi et al, 2021	-44.2	6.7	30	-41.9	9	30	16.2%	-0.29 [-0.79, 0.22]
**Total (95% CI)**			**227**			**225**	**100.0%**	**-0.46 [-0.88, -0.04]**

Heterogeneity: Tau² = 0.22; Chi² = 23.88, df = 5 (P = 0.0002); I² = 79%
Test for overall effect: Z = 2.15 (P = 0.03)

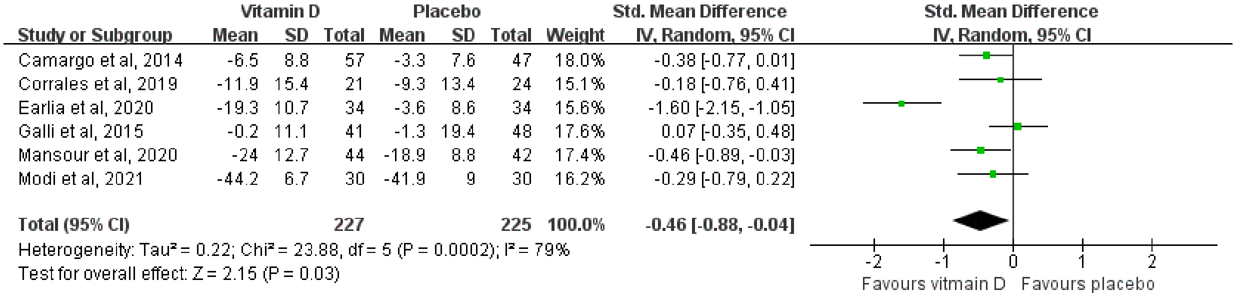

Figure S6. Meta analyses of change in serum 25(OH)D concentration before and after intervention

Study or Subgroup	Vitamin D Mean	Vitamin D SD	Vitamin D Total	Placebo Mean	Placebo SD	Placebo Total	Weight	Mean Difference IV, Random, 95% CI
**3.1.1 Asthma**								
Alansari et al, 2017	14.9	8.6	116	3.9	5.5	115	5.5%	11.00 [9.14, 12.86]
Baris et al, 2014	12.2	9.6	17	0	10.4	15	5.0%	12.20 [5.23, 19.17]
Bar Yoseph et al, 2015	12.3	7.3	19	1.4	9.1	19	5.2%	10.90 [5.65, 16.15]
DIVA, 2016	34.4	18.8	11	22.2	17.5	11	3.7%	12.20 [-2.98, 27.38]
Ducharme et al, 2019	9.6	23.1	23	4.3	25.7	24	3.9%	5.30 [-8.66, 19.26]
El-Korashi et al, 2021	16.9	4	23	2	4	23	5.5%	14.90 [12.59, 17.21]
ESDAC, 2021	6	25	111	0.3	16.6	107	5.2%	5.70 [0.09, 11.31]
Kerley et al, 2016	46.7	39	17	2.8	11.5	22	3.1%	43.90 [24.75, 63.05]
Majak et al, 2009	0.7	2.8	16	-1	3.2	17	5.5%	1.70 [-0.35, 3.75]
Majak et al, 2011	1.5	13.5	24	-3.2	15.1	24	4.8%	4.70 [-3.40, 12.80]
Swangtrakul et al, 2022	14	8.5	21	3.3	4.8	20	5.3%	10.70 [6.50, 14.90]
Tachimoto et al, 2016	1.8	8.5	54	3.1	8.7	35	5.4%	-1.30 [-4.97, 2.37]
VDKA, 2020	26.9	17.2	68	1.8	6.7	74	5.3%	25.10 [20.74, 29.46]
ViDASTA, 2021	19.8	9.3	28	2.1	9	28	5.3%	17.70 [12.91, 22.49]
**Subtotal (95% CI)**			**548**			**534**	**68.6%**	**11.24 [7.00, 15.47]**
Heterogeneity: Tau² = 52.56; Chi² = 189.94, df = 13 (P < 0.00001); I² = 93%								
Test for overall effect: Z = 5.20 (P < 0.00001)								
**3.1.2 Atopic dermatitis**								
Galli et al, 2015	49.9	68.3	41	0.4	22.9	48	2.7%	49.50 [27.61, 71.39]
Lara-Corrales et al, 2019	34.4	20.4	21	-1	19	24	4.3%	35.40 [23.83, 46.97]
Mansour et al, 2020	13.3	6	44	0.5	8.2	42	5.4%	12.80 [9.75, 15.85]
Modi et al, 2021	65.5	8.1	30	21	12.2	30	5.2%	44.50 [39.26, 49.74]
**Subtotal (95% CI)**			**136**			**144**	**17.7%**	**34.69 [13.52, 55.87]**
Heterogeneity: Tau² = 429.19; Chi² = 117.02, df = 3 (P < 0.00001); I² = 97%								
Test for overall effect: Z = 3.21 (P = 0.001)								
**3.1.3 Allergic rhinitis**								
Hassan et al, 2016	19.6	5.7	50	0.8	5.8	50	5.5%	18.80 [16.55, 21.05]
Jerzynska et al, 2016	46	6.2	21	10	3.3	24	5.4%	36.00 [33.04, 38.96]
Jerzynska et al, 2018	38.2	28.7	21	38.8	35.3	17	2.9%	-0.60 [-21.39, 20.19]
**Subtotal (95% CI)**			**92**			**91**	**13.8%**	**21.08 [5.99, 36.17]**
Heterogeneity: Tau² = 151.13; Chi² = 87.85, df = 2 (P < 0.00001); I² = 98%								
Test for overall effect: Z = 2.74 (P = 0.006)								
**Total (95% CI)**			**776**			**769**	**100.0%**	**16.84 [11.75, 21.93]**
Heterogeneity: Tau² = 122.20; Chi² = 656.57, df = 20 (P < 0.00001); I² = 97%								
Test for overall effect: Z = 6.48 (P < 0.00001)								
Test for subgroup differences: Chi² = 5.79, df = 2 (P = 0.06), I² = 65.4%								

Mean Difference IV, Random, 95% CI

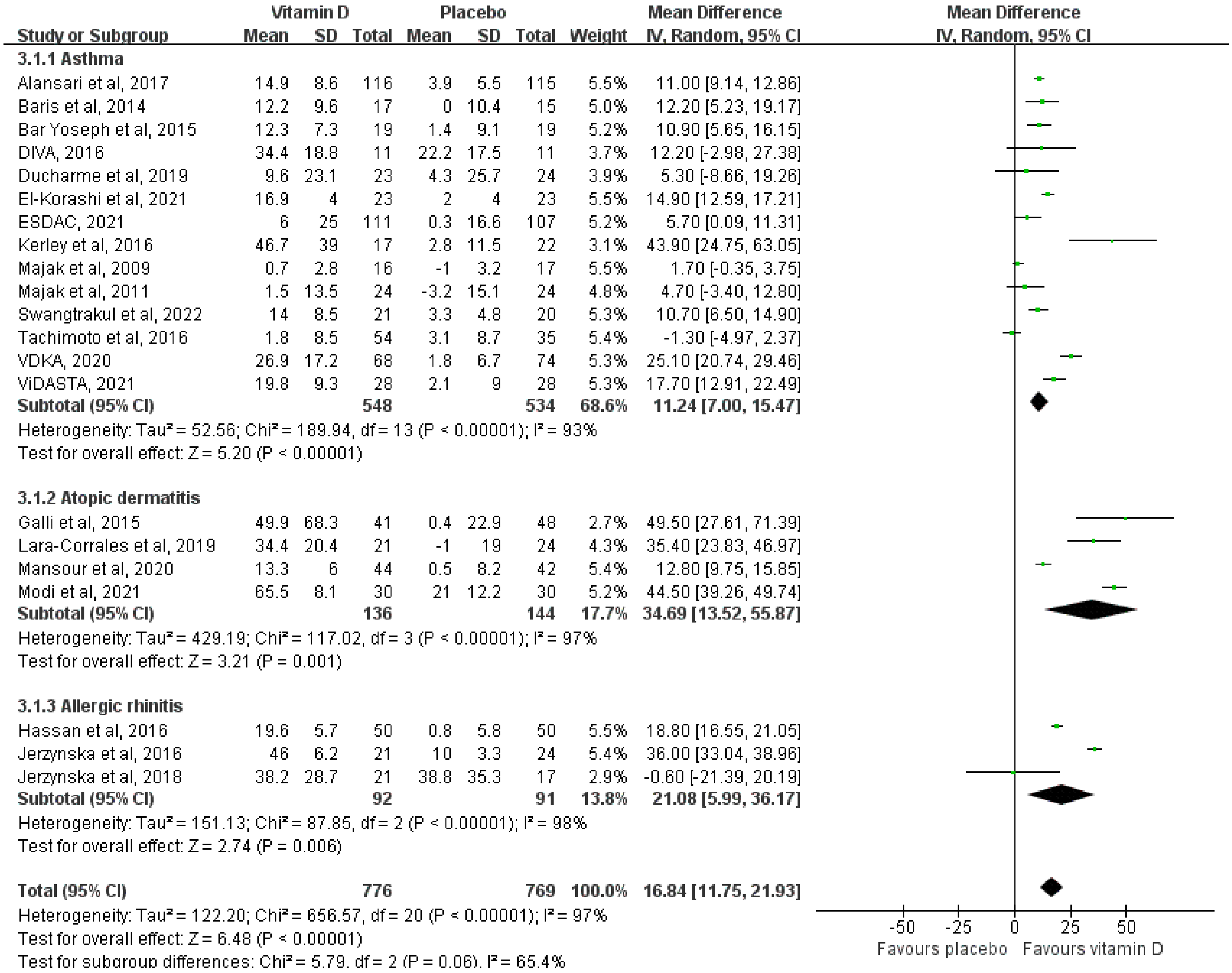

Figure S7. Meta analyses of post-intervention serum 25(OH)D concentration in vitamin D group

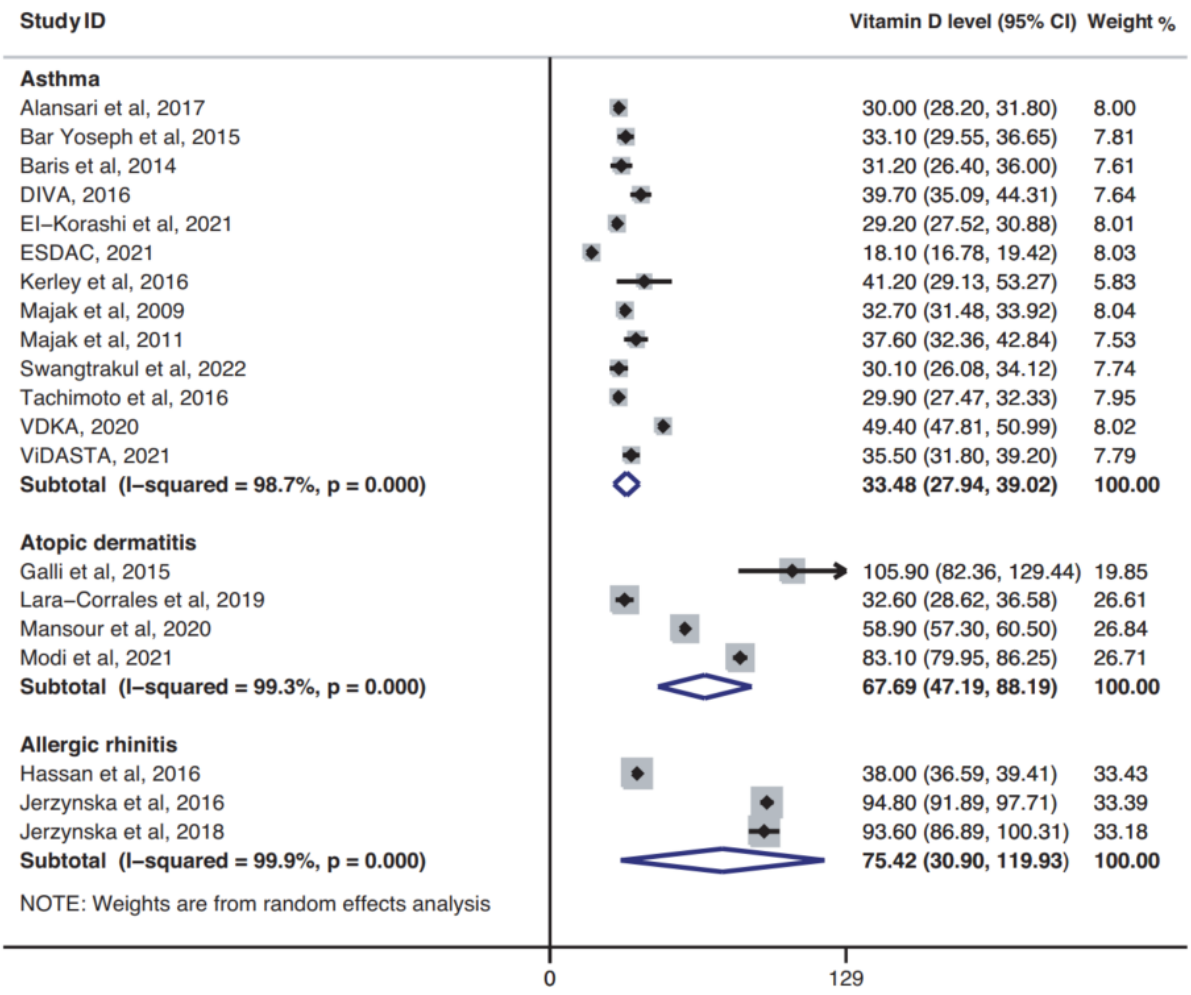

Appendix S1. Methods

Data extraction

We collect the following variables: 1) study general features: title, author, year of publication, number of patients included in the study; 2) participant general features: country, type of diseases, disease severity, age, gender, body-mass index (BMI), baseline serum 25(OH)D; 3) details of the intervention and control measures: dosage of vitamin D, frequency of administration, treatment duration, co-treatment; 4) study outcomes: primary and secondary outcomes. Dichotomous outcomes were extracted as the number of patients who had each outcome and the total number of patients. Continuous outcomes were extracted as sample size and mean (standard deviation, SD) or median (interquartile range, IQR) provided in the studies. The outcome recorded at the last visit was selected if the patients were followed many times.

Methods for imputation of missing standard deviations

We used published standard deviations (SDs), where available. When standard errors instead of SDs were presented, the former was converted to SDs. If both were missing, we estimated SDs from P values or confidence interval (CIs) according to the recommendations of the Cochrane Handbook for Systematic Reviews. We also estimated SDs from graphs when they were missing in tables or in text. If studies reported median, range and/or interquartile range, we used median to impute the missing mean and calculated SDs. If none of these options are viable, we imputed the missing SDs using pooled SDs from other studies included in our systematic review following the formula below [1]:

$$SD_{pooled} = \sqrt{\frac{\sum(n_i - 1)SD_I^2}{\sum(n_i - 1)}}$$

Risk of bias (RoB) assessment

Two groups of investigators (group 1 was QL and QZ; group 2 was ZW and YZ) independently assessed the RoB using the Cochrane collaboration risk of bias tool [1]. We assessed RoB according to the following domains: random sequence generation, allocation concealment, blinding of participants and personnel, blinding of outcome assessment, incomplete outcome data, selective outcome reporting, and other bias. Each domain was graded as 'low', 'high', or 'unclear' risk. Disagreement regarding RoB assessments was resolved by a third investigator (YC).

Quality of evidence assessment

We assessed the quality of the evidence with the Grading of Recommendations Assessment, Development and Evaluation (GRADE) approach for all outcomes [2]. We rated the quality of the evidence for each

outcome based on considerations of five factors that may lead to rating down the quality of evidence (risk of bias, inconsistency, imprecision, indirectness, and publication bias). The quality of the evidence is rated as 'high', 'moderate', 'low', or 'very low'. We performed the assessment using the GRADEpro software and generated a summary of findings table [3].

References

1.Higgins JPT, Thomas J, Chandler J, et al (editors). Cochrane Handbook for Systematic Reviews of Interventions. 2nd Edition. Chichester (UK): John Wiley & Sons, 2019.

2.Guyatt GH, Oxman AD, Vist GE, et al. GRADE: an emerging consensus on rating quality of evidence and strength of recommendations. BMJ. 2008;336(7650):924-926.

3.Guyatt GH, Oxman AD, Santesso N, et al. GRADE guidelines: 12. Preparing summary of findings tables-binary outcomes. J Clin Epidemiol. 2013;66(2):158-172.
